# Supramolecular covalency of halogen bonds revealed by NMR contact shifts in paramagnetic cocrystals

**DOI:** 10.1039/d5sc05769h

**Published:** 2025-10-02

**Authors:** Anagha Sasikumar, Jan Novotný, Jan Chyba, Libor Kobera, Radek Marek

**Affiliations:** a CEITEC – Central European Institute of Technology, Masaryk University Kamenice 5 CZ-62500 Brno Czechia radek.marek@ceitec.muni.cz; b Department of Chemistry, Faculty of Science, Masaryk University Kamenice 5 CZ-62500 Brno Czechia; c National Center for Biomolecular Research, Faculty of Science, Masaryk University Kamenice 5 CZ-62500 Brno Czechia; d Institute of Macromolecular Chemistry, Czech Academy of Sciences Heyrovského nám. 2 CZ-16200 Prague Czechia

## Abstract

Although supramolecular interactions such as halogen bonding are often classified as “non-covalent” interactions, computational methods have predicted that they possess a significant covalent component. In this article, we adopt a unique strategy that combines experimental solid-state NMR and relativistic DFT approaches to explore the electronic nature of previously postulated supramolecular covalency [Bora *et al.*, *Chem.–Eur. J.*, 2017, **23**, 7315]. Our approach involves the analysis of hyperfine interactions and hyperfine shifts in the NMR spectra of halogen-bonded cocrystals containing a paramagnetic transition-metal complex. We demonstrate that the hyperfine interaction pertaining to the paramagnetic transition-metal center and observed at the probed nucleus of the cocrystallized (halogen-bonded) molecule is governed by the Fermi-contact mechanism. This contact mechanism originates in “through-bond” spin transmission and, therefore, unequivocally reports on the electron sharing between the halogen-bonded molecules, *i.e.*, halogen-bond covalency.

## Abstract 2

A unique strategy integrating paramagnetic solid-state NMR spectroscopy and relativistic DFT is used to elucidate the nature of intermolecular halogen bonding. The Fermi-contact hyperfine mechanism emerges as a highly effective probe of this bonding.

## Graphical abstract

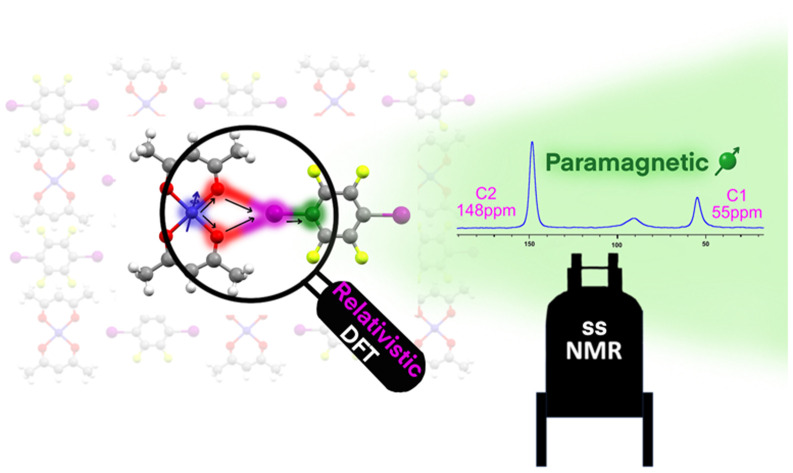

## 1 Introduction

Supramolecular interactions are the essence of life on Earth and the driving force in the creation of molecular assemblies and functional materials.^1–3^ Given their fundamental role in chemical and biological transformations,^4^ their nature is the subject of intense theoretical and experimental research. This includes diffraction analysis,^5,6^ spectroscopic methods,^7,8^ and theoretical approaches^9^ based on first principles. One of the most powerful spectroscopic methods is magnetic resonance, which studies the spin response of electrons (electron paramagnetic resonance, EPR) and nuclei (nuclear magnetic resonance, NMR). Although NMR spectroscopy is currently mainly used to analyze diamagnetic systems, its application to paramagnetic systems is gaining importance with the continuous development of instrumentation and theoretical description. Paramagnetic NMR spectroscopy (pNMR) is therefore currently an area of intense interest for a number of disciplines in chemistry, physics, biology, and medicine.^10^

In addition to standard parameters such as NMR shift and indirect nuclear spin–spin coupling, the temperature-dependent hyperfine (or Curie) shift is an extra quantity in NMR spectroscopy of open-shell systems. This shift originates from the hyperfine interaction between nuclear and electron spins and consists of three principal terms: Fermi-contact (FC), paramagnetic spin–orbit, and spin–dipolar.^11^ The FC term is based on spin transmission from the paramagnetic (metal) center to the observed ligand nucleus, whereas the other two terms operate “through space” over long distances (up to *ca.* 2 nm).^12^

At multiple-bond distances in molecules, the FC mechanism of electron-nucleus hyperfine coupling resembles that of indirect nuclear spin–spin coupling (*J*) in terms of the transmission of the spin polarization connected with electron exchange interactions.^13,14^ This equally applies to the transmission of spin polarization through a supramolecular (intermolecular) interaction, which depends on orbital interactions between the individual molecules. The FC term is thus an excellent indicator of the extent of electron sharing between atoms or molecules, *i.e.*, the covalency of bonding. It clearly applies to situations with intermolecular delocalization of a singly occupied molecular orbital (SOMO), as shown in the following section for supramolecular cocrystals.

In this article, we demonstrate how the experimental hyperfine shift is used to probe the covalency of halogen bonding (XB) in paramagnetic cocrystals. We note that this approach is generally applicable to a variety of supramolecular interactions in both solid and liquid states.

## 2 Results and discussion

### 2.1 Molecular arrangements in halogen-bonded cocrystals

**Fig. 1:**
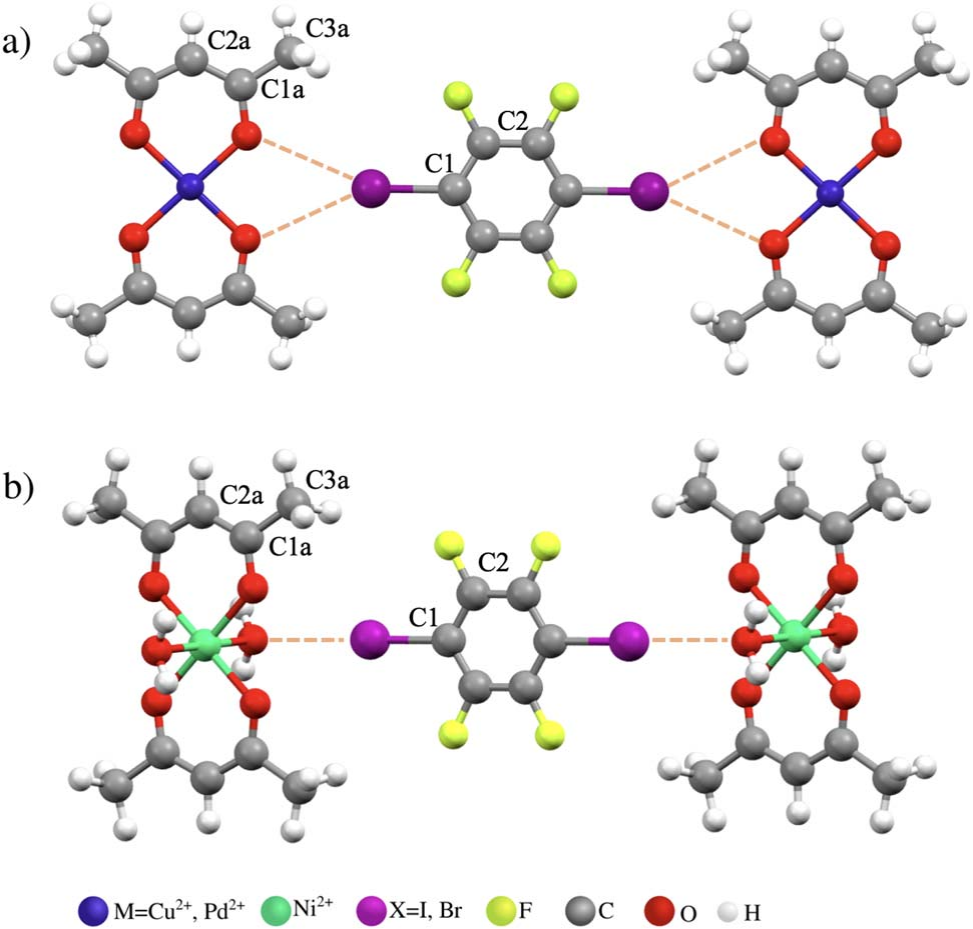
Cluster arrangements of (a) two molecules of TM acetylacetonate complex (1: M = Cu^2+^; 2: M = Pd^2+^) and one molecule of 1,4-dihalo-2,3,5,6-tetrafluorobenzene (I: X = I; Br: X = Br) and (b) two hydrated molecules of Ni^2+^ complex and one molecule of 1,4-diiodo-2,3,5,6-tetrafluorobenzene, 3I.

Cocrystals are formed of two or more molecular components held together by supramolecular (intermolecular) forces to form crystals of unique properties.^15^ These supramolecular interactions arise from the delicate balance of all interatomic forces such as electrostatics, covalency, and dispersion. The electrostatic component has previously been shown to be important for halogen and chalcogen bonds.^16^ However, it has also been demonstrated that the high-lying electron pair of the electron donor interacts with the low-lying vacant MO that involves a halogen or chalcogen in the electron acceptor.^17,18^ Applied to the system 1 investigated in this work (Fig. 1a), two lone pairs of electrons on two neighboring oxygen atoms of acetylacetonate (acac) ligands interact with σ*(I–C1) of the electron-deficient aromatic ring. This represents a classical orbital interaction between the occupied and vacant MOs forming a covalent contribution (electron sharing) to the halogen bond. As described in the Introduction, the covalency of the bond(s) forms a substance for spin transmission and the Fermi-contact mechanism of hyperfine shift in the NMR spectrum. In the next sections, we describe NMR experiments for 1I, 2I, 1Br, and 2Br and analyze hyperfine shifts using density functional theory (DFT).

### 2.2 NMR shifts for cocrystals 1 and 2

#### 2.2.1 Experimental ^13^C NMR spectroscopy

**Fig. 2:**
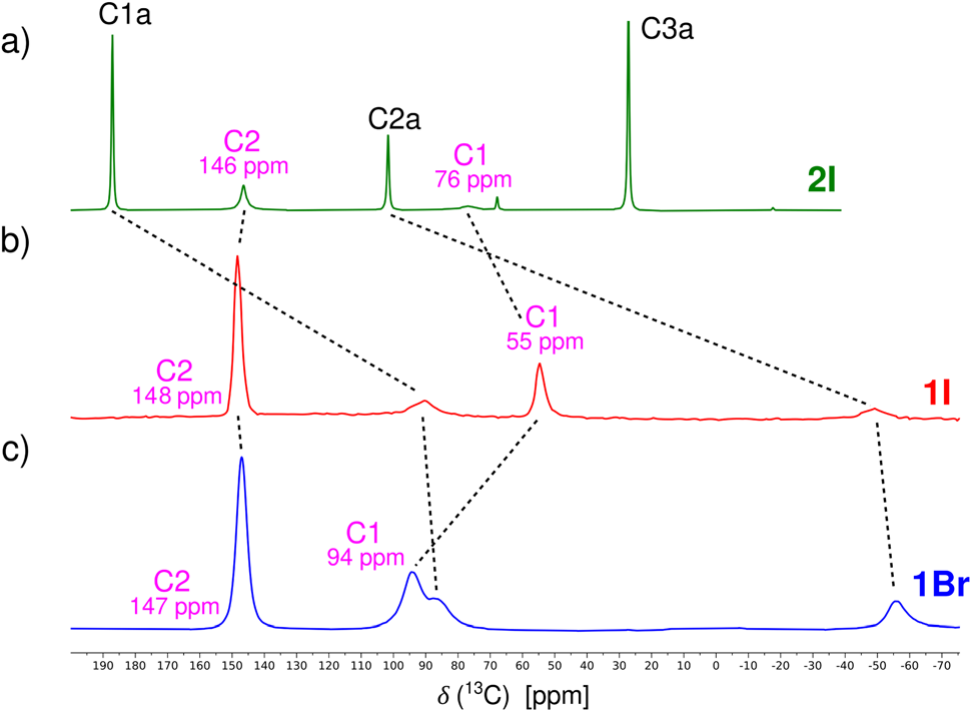
^13^C NMR spectra of systems (a) 2I, (b) 1I, and (c) 1Br recorded by using MAS techniques at laboratory temperature (see the SI).

First, paramagnetic cocrystal 1I with the central atom of Cu^2+^ has been measured using the ^13^C VF/MAS NMR technique (Fig. 2b). In the recorded NMR spectrum, four carbon resonances were observed (C3a not detected because of very fast paramagnetic relaxation).^19^ The two resonances of the acac ligand at +91 ppm (C1a) and −49 ppm (C2a) correspond relatively well to those previously reported for the pure compound 1 (+96 ppm and −67 ppm).^19^ The two carbon atoms C1 and C2 of halogenated aromatics resonate at +55 ppm and +148 ppm, respectively. To estimate their orbital (*δ*^orb^) and hyperfine (*δ*^HF^) contributions to total NMR shifts, we also recorded the ^13^C CP/MAS NMR spectrum of a cocrystal with its diamagnetic Pd^2+^ analog 2I (Fig. 2a).

The experimental ^13^C NMR shifts for compounds 1I (1Br) and 2I (2Br) are summarized in Table 1. Clearly, there is a difference of about 20 ppm in the NMR shift of C1 between 1I and 2I, whereas only a marginal difference has been observed for C2. Similarly, a 20 ppm difference has been observed for C1 between 2I and 2Br (Table 1). To interpret these differences unequivocally, we performed DFT calculations and analysis of ^13^C NMR shifts.

**Table 1:** Experimental ^13^C NMR shifts (in ppm) for systems 1I, 2I, 1Br, 2Br, and 3I measured by MAS techniques at laboratory temperature^a^

Cocrystal	C1	C2	C1a	C2a	C3a
1I (ref. 20)	55	148	91	−49	n.o.^b^
2I (ref. 21)	76	146	187	102	27
1Br (ref. 20)^c^	94	147	87	−56	n.o.^b^
2Br^c^	95	145	187	101	26
3I (ref. 20)^c^	77	148	277	136	805

a For the experimental setup and ^13^C NMR spectra (Fig. S7–S9), see the SI.

b Not observed.

c This work, CCDC No. 2472007 for 1Br, 2472009 for 2Br, and 2472008 for 3I.

#### 2.2.2 DFT calculations

Theoretical values of ^13^C NMR shifts were calculated using the DFT methodology implemented in the ADF program.^22^ The calculations were performed on model structures obtained from X-ray diffraction analysis (see the SI).^20,21^ Initially, we performed the calculations at the scalar-relativistic level (1c ZORA; **g**-tensor at 2c) neglecting spin–orbit coupling. The orbital shifts (*δ*^orb^) for clusters 1I (Cu^2+^) and 2I (Pd^2+^), and hyperfine shifts (*δ*^HF^) for open-shell system 1I are summarized in Table 2.

**Table 2:** ^13^C NMR shifts of C1 and C2 (in ppm) for 1I and 2I calculated at the 1c DFT level (**g**-tensor at the 2c level)^a^

System	Atom	*δ* ^orb^	*δ* ^HF^(FC)^b^	Total
1I	C1	114	−16	98
	C2	147	+1	148
2I	C1	110	—	110
	C2	148	—	148

a ZORA, PBE50/TZ2P, 298 K. To calculate *δ*^orb^ at the ZORA level, Cu^2+^(↑) was replaced by Ni^2+^(↑↓) in 1I.^23^ For the computational details and full set of data (Table S2), see the SI.

b The value was obtained by averaging *δ*^HF^ for all spin states.

The calculated ^13^C NMR shifts are referenced relative to benzene, the secondary reference used (128 ppm). Hyperfine shifts were obtained from the calculated EPR parameters (electronic **g**-tensor, hyperfine coupling **A**-tensor) using the *PNMRShift* program.^24^ The hyperfine shifts of all carbon atoms are dominated by the FC terms (for a full set of data, see the SI) and the pseudo-contact contributions are negligible (<0.1 ppm).

To report on the covalency of the supramolecular halogen bond between two oxygen atoms in Cu(acac)_2_ and iodine in C_6_F_4_I_2_ in the cocrystal, we analyze the FC contribution to the ^13^C NMR shift of atom C1 directly attached to iodine (Fig. 1). The FC contribution originates in the spin transmission from the paramagnetic metal to the probed carbon. This is facilitated by covalent contribution (electron sharing) to the supramolecular bonding between O and I atoms. The PBE50-calculated hyperfine (FC) contribution of −16 ppm is somewhat underestimated compared to the experimental difference of −21 ppm between 2I and 1I (however, the PBE0 value of −26 ppm is overestimated due to the delocalization error,^25^ see Fig. S12 in the SI). Note that the FC contribution for C2 is marginal as a result of inefficient spin transmission to this more distant atom.

#### 2.2.3 Spin–orbit effects on NMR shifts

To determine the effects of spin–orbit (SO) coupling on both the orbital and hyperfine (particularly FC term) shifts of C1 and C2 in 1I and 1Br, we repeated the calculations with the inclusion of SO coupling (2c SO-ZORA). First, the orbital contributions (*δ*^orb^) have been approximated by the NMR shifts for their corresponding light diamagnetic analogs (Table 3).^23^ Second, we calculated ^13^C NMR shifts in heavier diamagnetic Pd^2+^ system 2I that was measured experimentally. The *δ*^orb^ and *δ*^HF^ of C1 and C2 calculated at the 2c DFT level and those obtained experimentally are summarized in Table 3.

**Table 3:** ^13^C NMR shifts (in ppm) of C1 and C2 for systems 1I, 1Br, 2I, 2Br, and 3I calculated at the 2c DFT level^a^ and obtained experimentally

System	Atom	*δ* ^orb^	*δ* ^HF^(FC)^b^	Total	Experimental
1I	C1	78	−16	62	55
	C2	147	+1	148	148
1Br	C1	102	−9	93	94
	C2	143	0	143	147
2I	C1	76	—	76	76
	C2	147	—	147	146
2Br	C1	104	—	104	95
	C2	145	—	145	145
3I	C1	73	−2	71	77
	C2	145	0	145	148

a SO-ZORA, PBE50/TZ2P, 298 K. To calculate *δ*^orb^ at the SO-ZORA level, Cu^2+^(↑) was replaced by Ni^2+^(↑↓) in 1, and Ni^2+^(↑↑) was replaced by Zn^2+^(↑↓) in 3.^23^ For the computational details and full set of data (Table S3), see the SI.

b The value was obtained by averaging *δ*^HF^ for all spin states.

By comparing the orbital shift (*δ*^orb^) of the C1 atom in 2I calculated at the 1c (ZORA) and 2c (SO-ZORA) DFT levels of theory, a significant effect of SO coupling has been identified for the C1 atom directly bound to iodine. This is attributed to the well-known SO-HALA effect^26^ pertinent to heavy iodine. The SO-HALA shielding in aromatic systems typically amounts to approximately 30–50 ppm for ^13^C–I and 10–20 ppm for ^13^C–Br,^27^ which fits well with our present observations (−36 ppm for 1I). However, spin–orbit coupling may also influence the FC contribution to the NMR shift. Therefore, hyperfine shifts for 1I were calculated at the 2c DFT level (Table 3) and compared with 1c DFT values (Table 2). Clearly, the spin–orbit contribution to the FC shift is negligible. Before extending our analysis to a more structural arrangement, we performed an analysis of spin density and the mechanism of spin transmission in 1I.

#### 2.2.4 FC mechanism of hyperfine shift and analysis of spin density

**Fig. 3:**
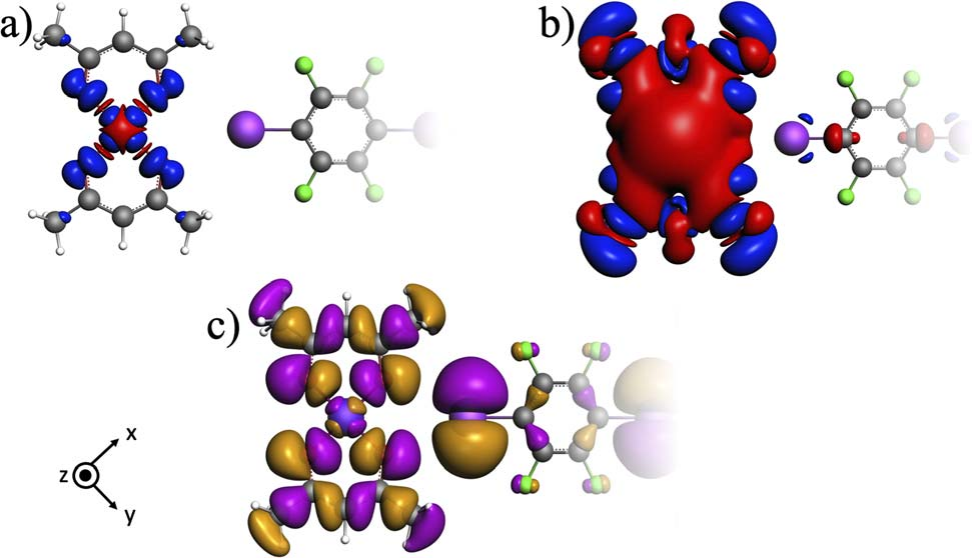
Visualization of the spatial distribution of spin density (α in blue and β in red) at the isovalues of (a) 0.001 au and (b) 0.00001 au, and (c) SOMO for system 1I.

Spin transmission from the metal center to the organic moiety in the cocrystal can occur through the spin delocalization or spin polarization mechanism.^11,13^ To analyze hyperfine interactions and electron sharing through the halogen bond in detail, the distribution of the spin density in system 1I was calculated and visualized in Fig. 3. As the SO contribution to the FC shift of C1 is negligible, we resorted to the calculations at the scalar-relativistic DFT level.

The distribution of α-spin density in the plane of acac ligands (Fig. 3a in blue) points to the spin delocalization of the singly occupied molecular orbital (SOMO, d_*x*^2^−*y*^2^_-type shown in Fig. 3c). The spin density is delocalized to the oxygen atoms of acac ligands and further propagated to the p-type in-plane orbital at iodine (Fig. 3b) and weakly also to the C1–C2 bond in the organic moiety. This supramolecular spin transmission is facilitated by the partial covalency of the O⋯X halogen bond and leads to a weak addition of α-density at the I and C2 atoms causing an excess of β-density at C1 due to the spin-polarization mechanism.^13^ Clearly, spin transmission through the halogen bond is related to its bonding character, which is thoroughly analyzed in the next section.

### 2.3 Character of the halogen bond in 1 and 2

#### 2.3.1 Delocalization index, QTAIM

To quantify the electron sharing between the atomic basins of oxygen and halogen in system 1I*vs.*1Br, QTAIM analysis^28^ of the delocalization index (DI) was performed using the ADF software^22^ (for Computational details, see the SI). The DI,^29^ derived from the exchange–correlation density, is the covariance of the joint probability distribution for the number of electrons in two atomic basins, representing the electron sharing between them. The DI for each of the two equivalent O⋯X bonds in 1I and 1Br was calculated to be 0.06 a.u. and 0.05 a.u., respectively. Taking into account bifurcation of the bonding, we arrive at values of 0.12 a.u. (1I) and 0.10 a.u. (1Br) for supramolecular (intermolecular) DIs. Although these numbers indicate weaker interactions compared to the DI of 0.44 a.u. for the strong halogen bond between ammonia and iodofluoride (see the SI), they point to a significant covalent contribution. Together with the dominant electrostatic components, these covalent contributions present for both halogens of the 1,4-dihalo-2,3,5,6-tetrafluorobenzene molecule are sufficient to force both molecules into the layer arrangement in the cocrystal (see Fig. 1).

#### 2.3.2 Energy decomposition analysis (EDA) and natural orbitals for chemical valence (NOCV) analysis

The energy components of the bonding were analyzed in detail using Energy Decomposition Analysis (EDA) based on the Morokuma–Ziegler scheme implemented in the ADF package.^22,30^ The electrostatic (Δ*E*_Es_), Pauli repulsion (Δ*E*_Pauli_), orbital (Δ*E*_Orb_), and dispersion (Δ*E*_Disp_) contributions to the total interaction energy (Δ*E*_Int_) for the Cu(acac)_2_ and C_6_F_4_X_2_ fragments in 1I and 1Br at the scalar-relativistic level are summarized in Table 4.

**Table 4:** Energy decomposition analysis of interaction between Cu(acac)_2_ and C_6_F_4_X_2_ in the cocrystals 1I, 1Br, and 3I (Δ*E* in kcal mol^−1^). The interaction energies calculated for the clusters shown in Fig. 1a were each divided by 2 to represent a single bifurcated halogen bond^a^

System	Δ*E*_Es_	Δ*E*_Pauli_	Δ*E*_Orb_	Δ*E*_Disp_	Δ*E*_Int_
1I	−7.0	+4.3	−1.9	−3.4	−7.9
1Br	−4.3	+2.4	−1.8	−2.8	−6.5
3I	−7.2	+7.5	−3.2	−4.2	−7.0

a For comparison with the previously reported values, see the SI.

The total interaction energies indicate a stronger bifurcated halogen bond for 1I (−7.9 kcal mol^−1^) compared to 1Br (−6.5 kcal mol^−1^), consistent with the predictions from hyperfine contributions of NMR shifts. EDA analysis reveals a dominant electrostatic interaction in stabilization of the cocrystal for both systems. However, the orbital contribution, which indicates the electron sharing between the fragments, suggests a significant covalent character of the halogen bonds which accounts for approximately 25% of the total interaction energy (this is further supported by the calculated second-order stabilization energies for 1I and 1Br as summarized in Table S7).

##### 2.3.2.1 Natural orbitals for chemical valence

**Fig. 4:**
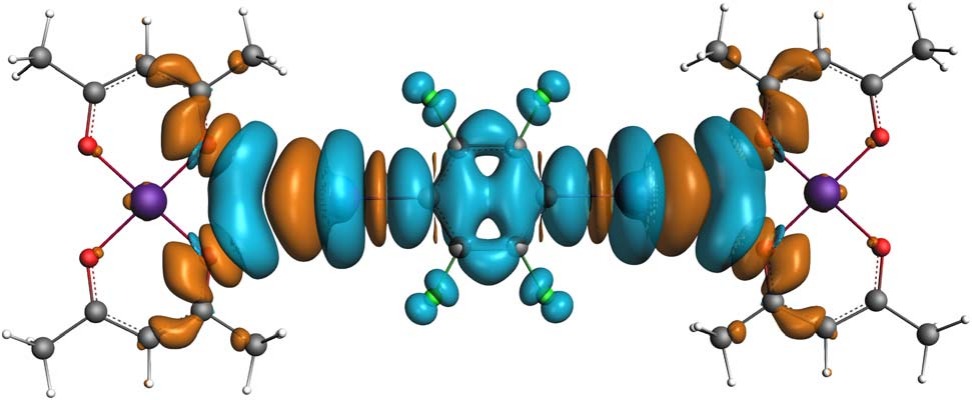
Electron deformation density (EDD) in system 1I (isovalue 0.00005 au) representing the sum of the four most important NOCV channels. Concentration and depletion of electron density shown in cyan and orange, respectively.

In the next step, an EDA-NOCV analysis^31,32^ was performed focusing on the orbital interactions between the donor and acceptor of the halogen bond. The sum of four NOCV channels involved in charge transfer from four *n*_O_ to two 
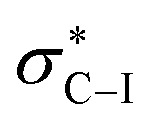
 in the molecular cluster 1I is shown in Fig. 4. This NOCV sum contributes to the orbital component of the interaction energy by 2.0 kcal mol^−1^ (1.0 kcal mol^−1^ per bifurcated halogen bond) for system 1I thus covering more than 50% of its Δ*E*_Orb_.

#### 2.3.3 Reduced coupling constants

As an alternative descriptor of electron sharing, we analyzed the reduced coupling constants (*K*, independent of the magnetogyric ratios of the coupled nuclei) between acac oxygens and halogen atoms, halogens and carbons C1, and long-range couplings between oxygens and C1 in 2I and 2Br (see Table S5 in the SI). Clearly, neighbor-atom couplings through halogen bonds are sizable, but long-range *K*_O–C1_ interactions are also non-negligible. Despite the longer distances of both O⋯X and X–C1 in 2I, the reduced coupling constant (*K* = 5.1 × 10^19^ kg m^−2^ s^−2^ A^−2^) is greater compared to that for 2Br (*K* = 2.8 × 10^19^ kg m^−2^ s^−2^ A^−2^). This is additional support for the stronger covalent component of the halogen bond identified in 2I compared to that in 2Br.

### 2.4 Hyperfine coupling pathways, cocrystal 3I

**Fig. 5:**
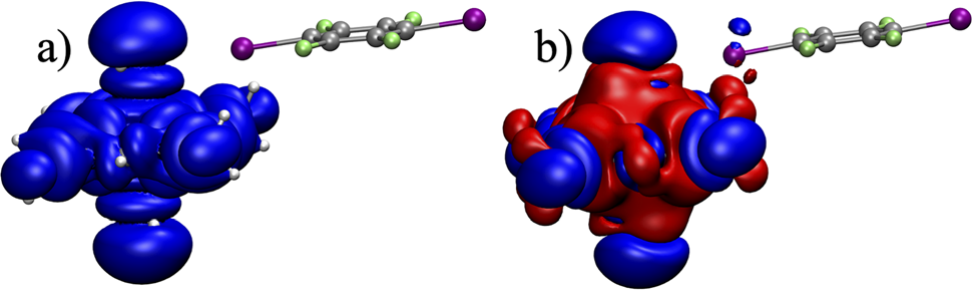
Visualization of the spatial distribution of spin density (α in blue and β in red, isovalue of 0.00001) for system 3I obtained using (a) restricted and (b) unrestricted scalar-relativistic DFT calculations.

To explore different crystal arrangements, we also analyzed a cocrystal of Ni(acac)_2_(H_2_O)_2_ with C_6_F_4_I_2_ (3I, 1b). The total interaction energy for 3I is comparable to that of 1I (Table 4). However, a very small *δ*^HF^ for the C1 atom (−2 ppm, Table 3) indicates a different mechanism of hyperfine interaction in 3I derived from the delocalization and polarization pathways (for detailed analyses and comparisons, see the SI). In system 3I, the spin density is efficiently transmitted to the H_2_O ligand in the axial position by the delocalization mechanism of one of the two SOMOs (for restricted DFT calculation of the spin density, see Fig. 5a). In contrast to 1I, there is a vanishingly small direct delocalization of SOMO to iodine. The spin polarization enabled in the unrestricted DFT calculation generates the α- and β-spin density above and below, respectively, the plane of the halogenated aromatic system (Fig. 5b). Note that this spin polarization involves two transmission pathways through the oxygen atoms of both the water and the acac ligands. The net effect results in a small total spin density in the π-space of iodine (out of plane). An additional spin-polarization step is required to generate in-plane spin density transmitted to the s atomic orbital of C1 that is essential for the FC mechanism. This process is rather inefficient and results in only a marginal contribution of the FC mechanism to the hyperfine shift of C1 in 3I. This contrasts with 1I, where the SOMO is directly in-plane delocalized to the aromatic systems (*vide supra*).

Clearly, despite the similar strength of the halogen bonding to the oxygen of the H_2_O ligand in 3I and the acac oxygens in 1I (Table 4), the FC mechanisms and hyperfine shifts of the C1 atoms are strikingly different. The results indicate that this descriptor can only be used directly in similar bonding situations (*e.g.*, 1I*vs.*1Br) or with the interpretative power of the accompanying theoretical calculations and analyses. However, it still represents a probe that is more sensitive (tens or even hundreds of parts per million) to report on covalency^18^ than secondary NMR shifts (a few ppm) induced by weak contacts between diamagnetic supramolecular components.^33^

## 3 Conclusion

This work provides solid support for classifying the halogen bond (XB) as a supramolecular interaction with a non-negligible covalent component. The phenomenon has previously been postulated as supramolecular covalency.^18^ In our unprecedented approach, we used the Fermi-contact mechanism of hyperfine interaction and hyperfine shift in paramagnetic NMR spectroscopy to probe electron sharing between two components of the cocrystal (connected *via* a bifurcated XB). We analyzed systems of paramagnetic Cu^2+^ and diamagnetic Pd^2+^ complexes of acetylacetonate ligands with *para*-C_6_F_4_X_2_, X = I, Br using solid-state ^13^C NMR spectroscopy and relativistic DFT calculations. We have shown that the contact mechanism (FC term) is the only source of the hyperfine shift (*δ*^HF^) of the C1 atom of the XB donor from the cocrystallized paramagnetic XB acceptor. This has been supported by analyzing the spin-transmission pathways through the bifurcated O⋯X halogen bond. Although the spin–orbit effects significantly varied the orbital shifts of C1, they were found to have a negligible impact on the hyperfine shifts. Our further analysis of the XBs indicates a somewhat lower degree of electron sharing between the XB donor and the acceptor in the case of 1Br compared to 1I, correlating with the smaller hyperfine shifts for the former. We also explored hyperfine coupling pathways in the paramagnetic Ni^2+^ complex with two axially coordinated water molecules, revealing that the FC mechanism crucially depends on the efficiency of the spin transmission. This supramolecular FC contribution represents a probe for detecting supramolecular contact more sensitively than the induced NMR shift in the diamagnetic system. Hence, our findings demonstrate that the Fermi-contact contribution to the hyperfine shift in paramagnetic NMR spectra serves as a highly sensitive indicator of supramolecular covalency. However, this phenomenon warrants further systematic investigation, and research in this direction is currently ongoing in our laboratory.

## Author contributions

AS: theoretical calculation, data curation, visualization, writing – original draft, review, and editing; JN: methodology, data curation, resources, writing – review and editing; JC: experimental investigation, data curation, writing – review and editing; LK: experimental investigation, data curation, writing – review and editing; RM: conceptualization, supervision, project administration, resources, investigation, writing – original draft, review, and editing.

## Conflicts of interest

There are no conflicts to declare.

## Supplementary Material

SC-016-D5SC05769H-s001

SC-016-D5SC05769H-s002

SC-016-D5SC05769H-s003

## Data Availability

The NMR spectra have been deposited at Mendeley Data and can be accessed *via*https://data.mendeley.com/datasets/54yjj55rt3/1 (DOI: https://doi.org/10.17632/54yjj55rt3.1). The computational results are available in the ioChem-BD repository and can be accessed *via*https://doi.org/10.19061/iochem-bd-6-589. CCDC 2472007–2472009 contain the supplementary crystallographic data for this paper.^34*a*–*c*^ The data that support the findings of this study are available in the supplementary information (SI). Supplementary information: experimental and theoretical methods, XRD, NMR, and DFT data. See DOI: https://doi.org/10.1039/d5sc05769h.
