# Supplementary figures and images for: Supramolecular covalency of halogen bonds revealed by NMR contact shifts in paramagnetic cocrystals
Source: Chem Sci. 2025 Oct 2;16(43):20239–44. doi: 10.1039/d5sc05769h (PMC12516516; doi:10.1039/d5sc05769h)

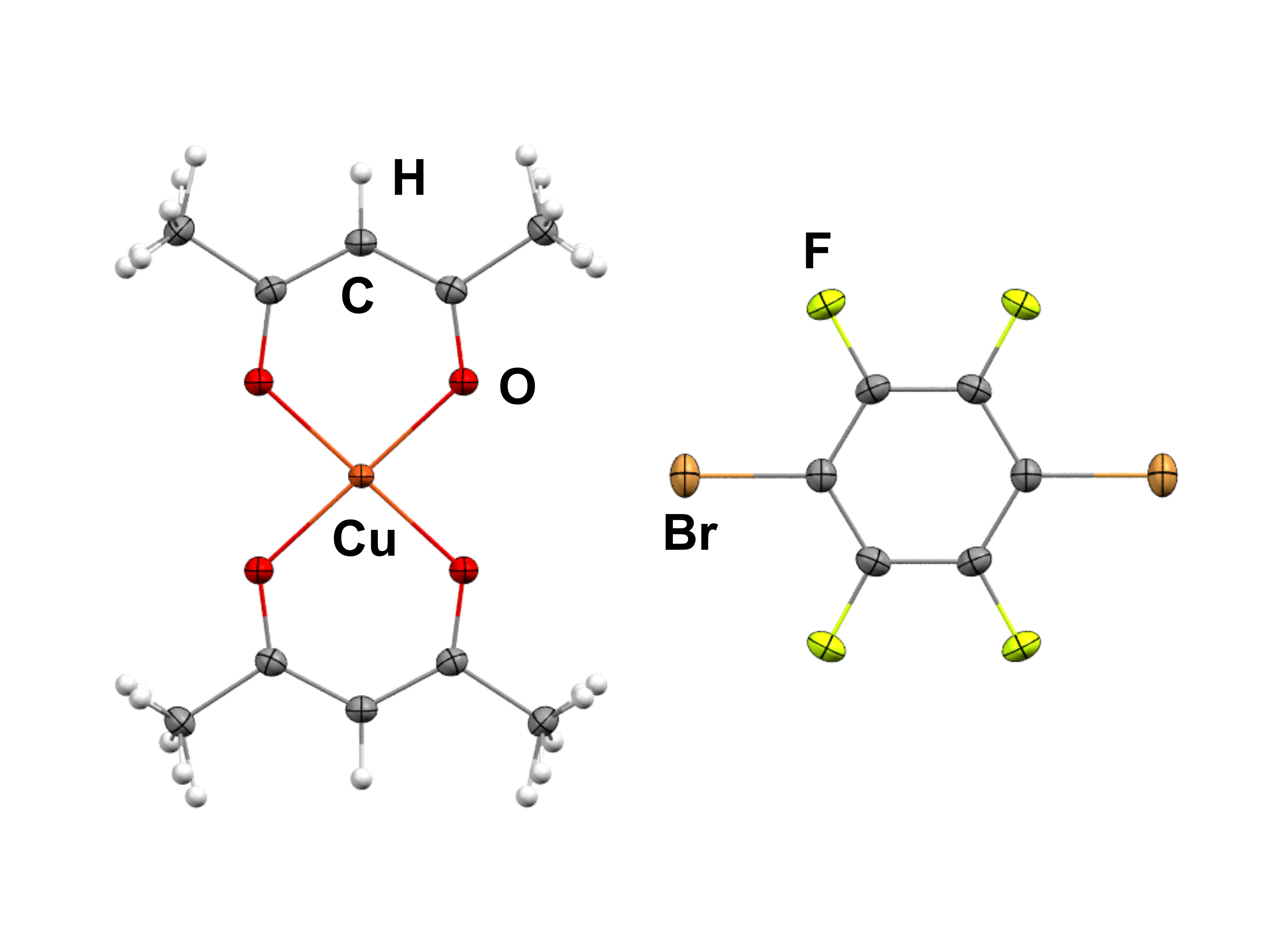

Supplement: SC-016-D5SC05769H-s001 [file SC-016-D5SC05769H-s001.zip › ESI Figures/FigureS1.png]

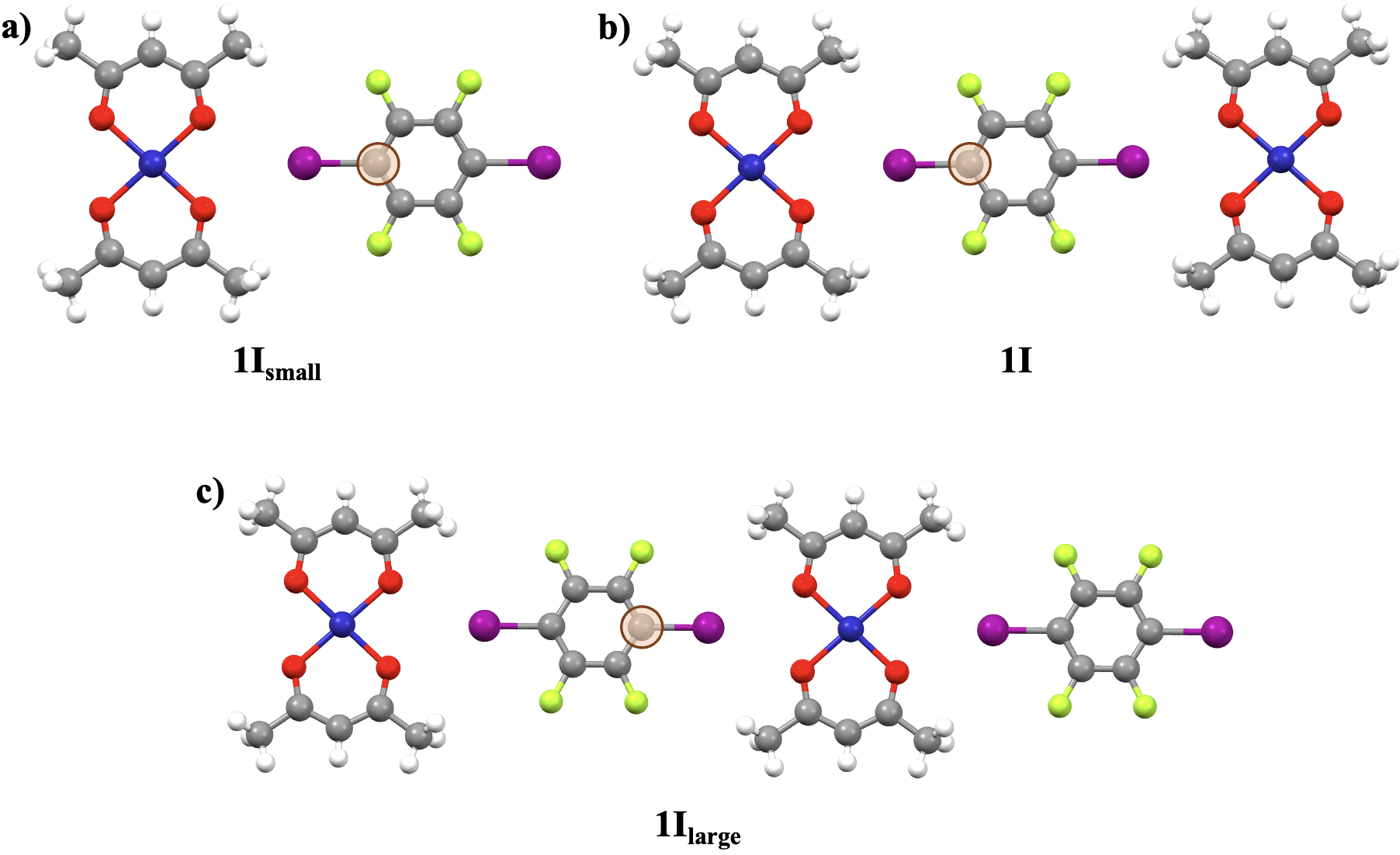

Supplement: SC-016-D5SC05769H-s001 [file SC-016-D5SC05769H-s001.zip › ESI Figures/FigureS10.png]

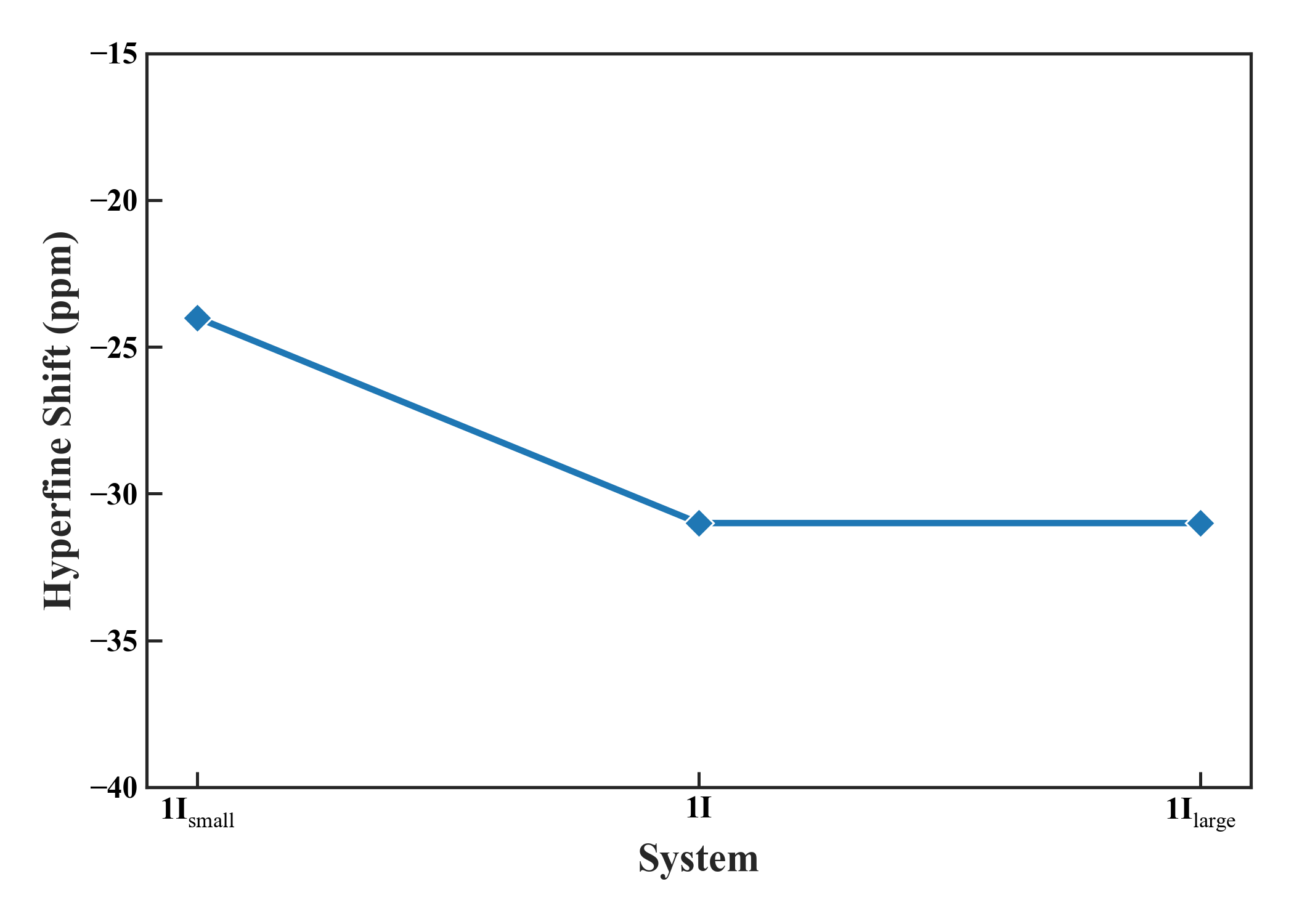

Supplement: SC-016-D5SC05769H-s001 [file SC-016-D5SC05769H-s001.zip › ESI Figures/FigureS11.png]

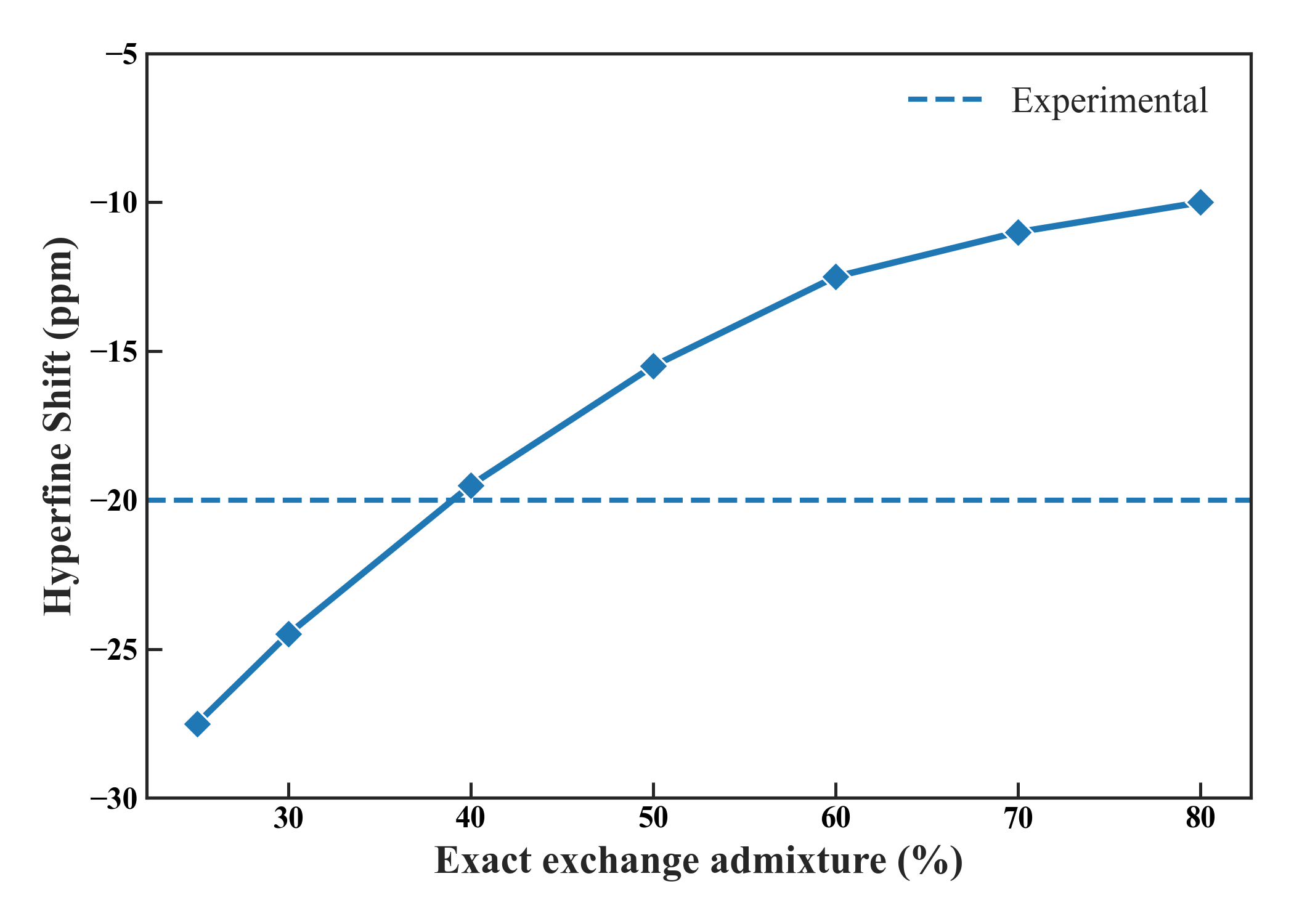

Supplement: SC-016-D5SC05769H-s001 [file SC-016-D5SC05769H-s001.zip › ESI Figures/FigureS12.png]

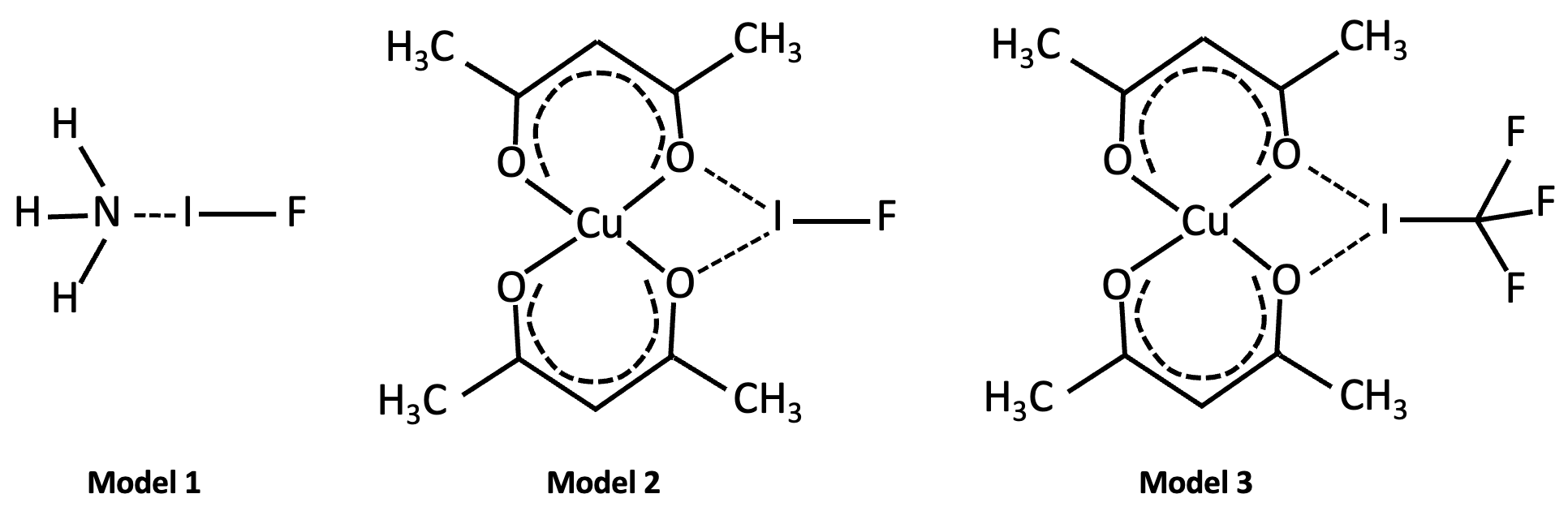

Supplement: SC-016-D5SC05769H-s001 [file SC-016-D5SC05769H-s001.zip › ESI Figures/FigureS13.png]

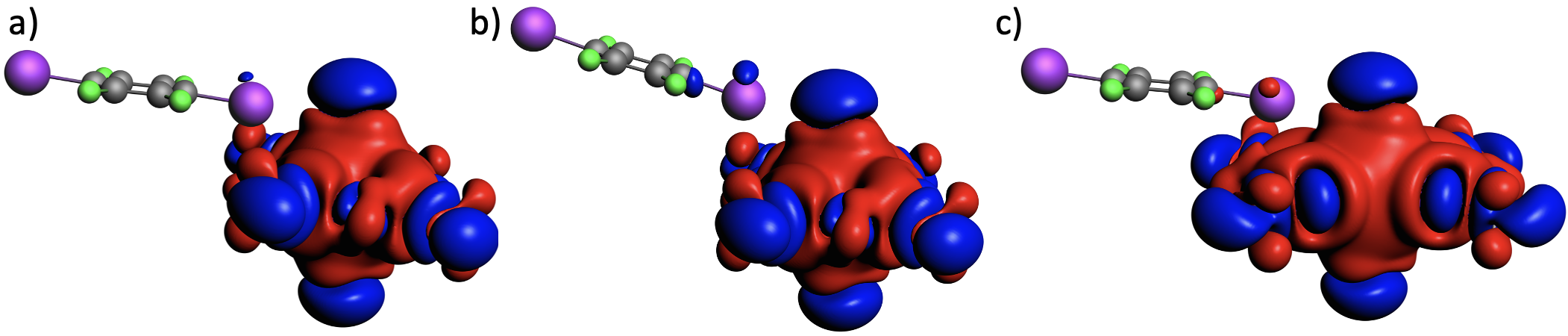

Supplement: SC-016-D5SC05769H-s001 [file SC-016-D5SC05769H-s001.zip › ESI Figures/FigureS14.png]

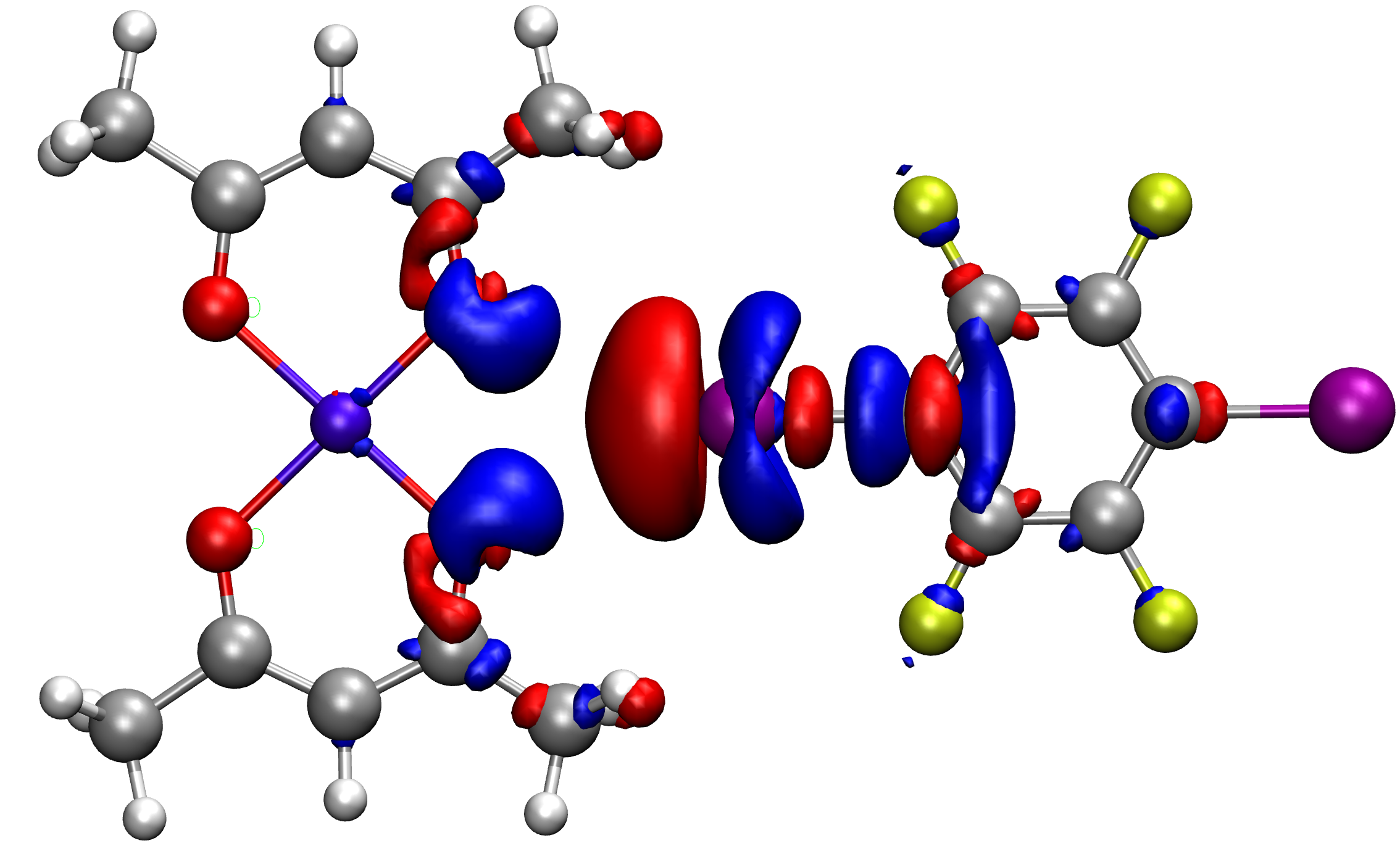

Supplement: SC-016-D5SC05769H-s001 [file SC-016-D5SC05769H-s001.zip › ESI Figures/FigureS15.png]

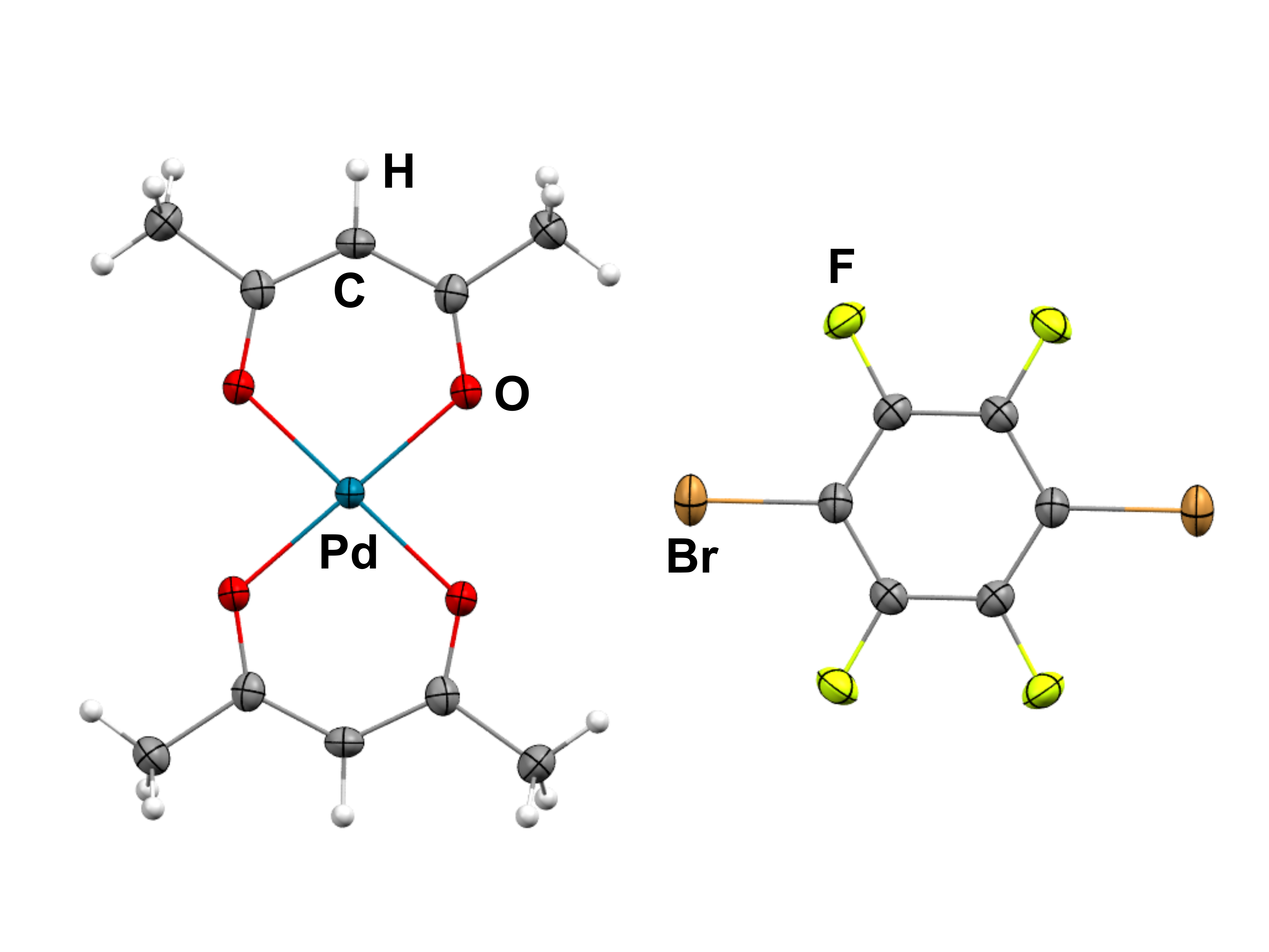

Supplement: SC-016-D5SC05769H-s001 [file SC-016-D5SC05769H-s001.zip › ESI Figures/FigureS2.png]

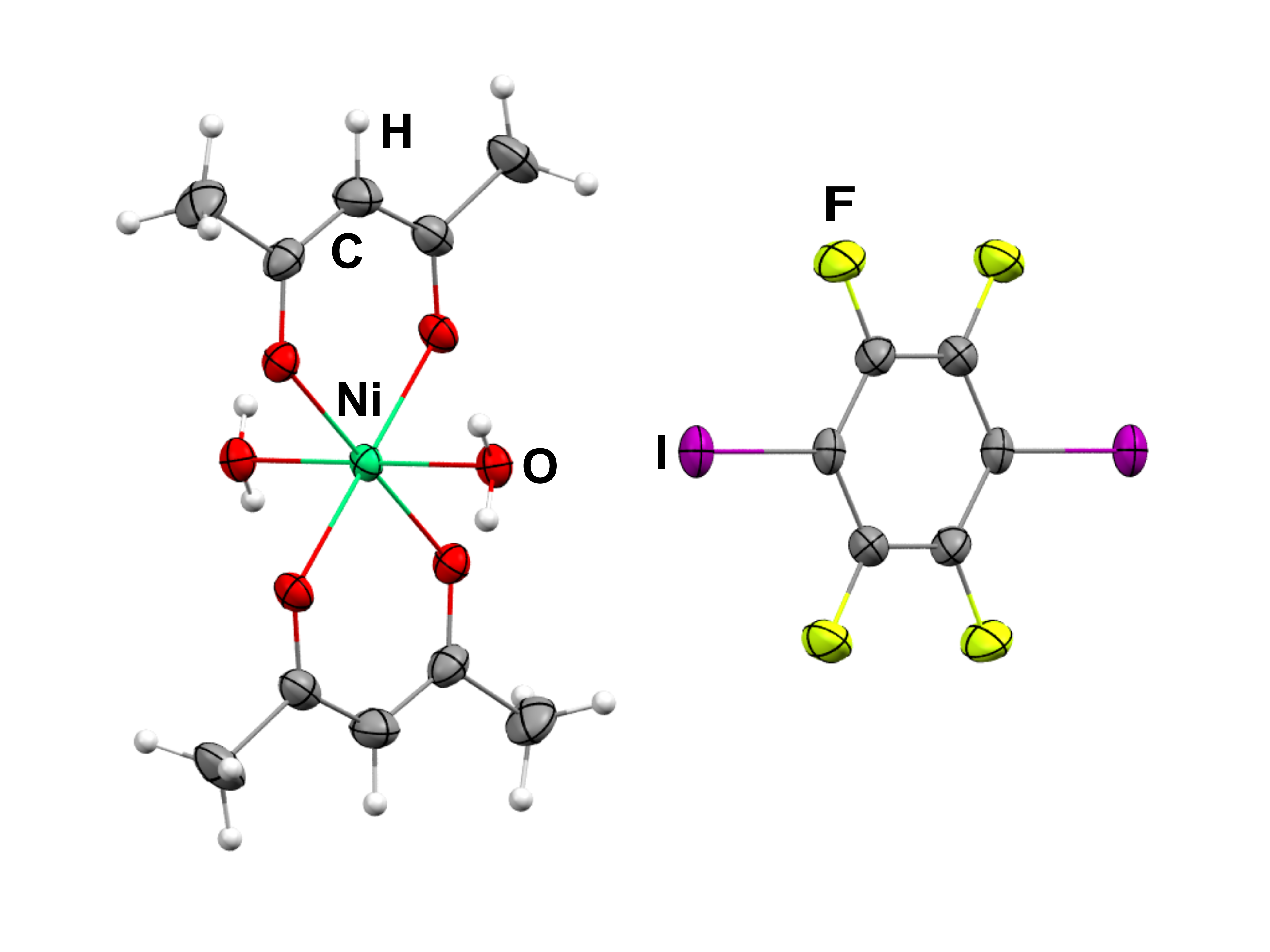

Supplement: SC-016-D5SC05769H-s001 [file SC-016-D5SC05769H-s001.zip › ESI Figures/FigureS3.png]

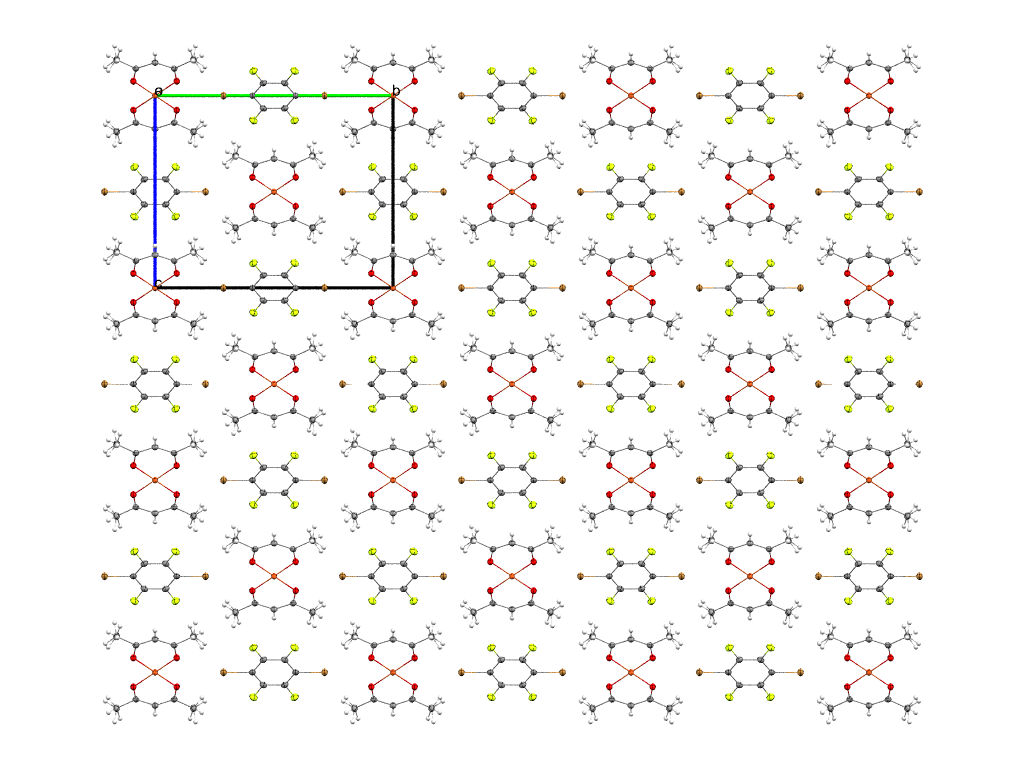

Supplement: SC-016-D5SC05769H-s001 [file SC-016-D5SC05769H-s001.zip › ESI Figures/FigureS4.png]

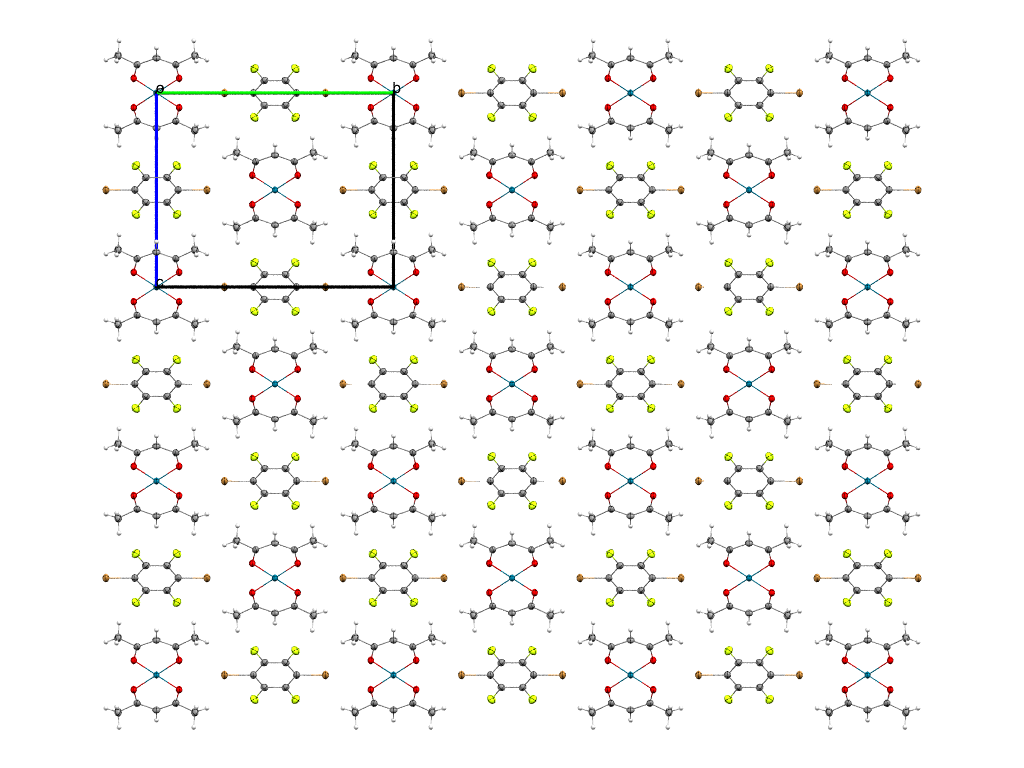

Supplement: SC-016-D5SC05769H-s001 [file SC-016-D5SC05769H-s001.zip › ESI Figures/FigureS5.png]

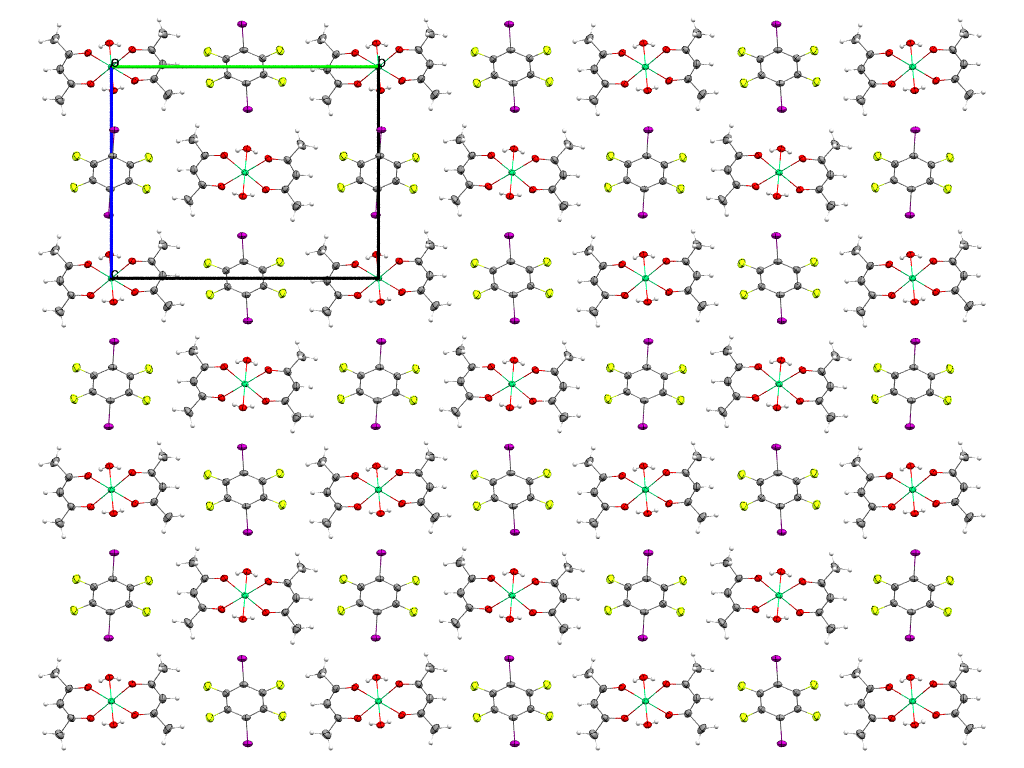

Supplement: SC-016-D5SC05769H-s001 [file SC-016-D5SC05769H-s001.zip › ESI Figures/FigureS6.png]

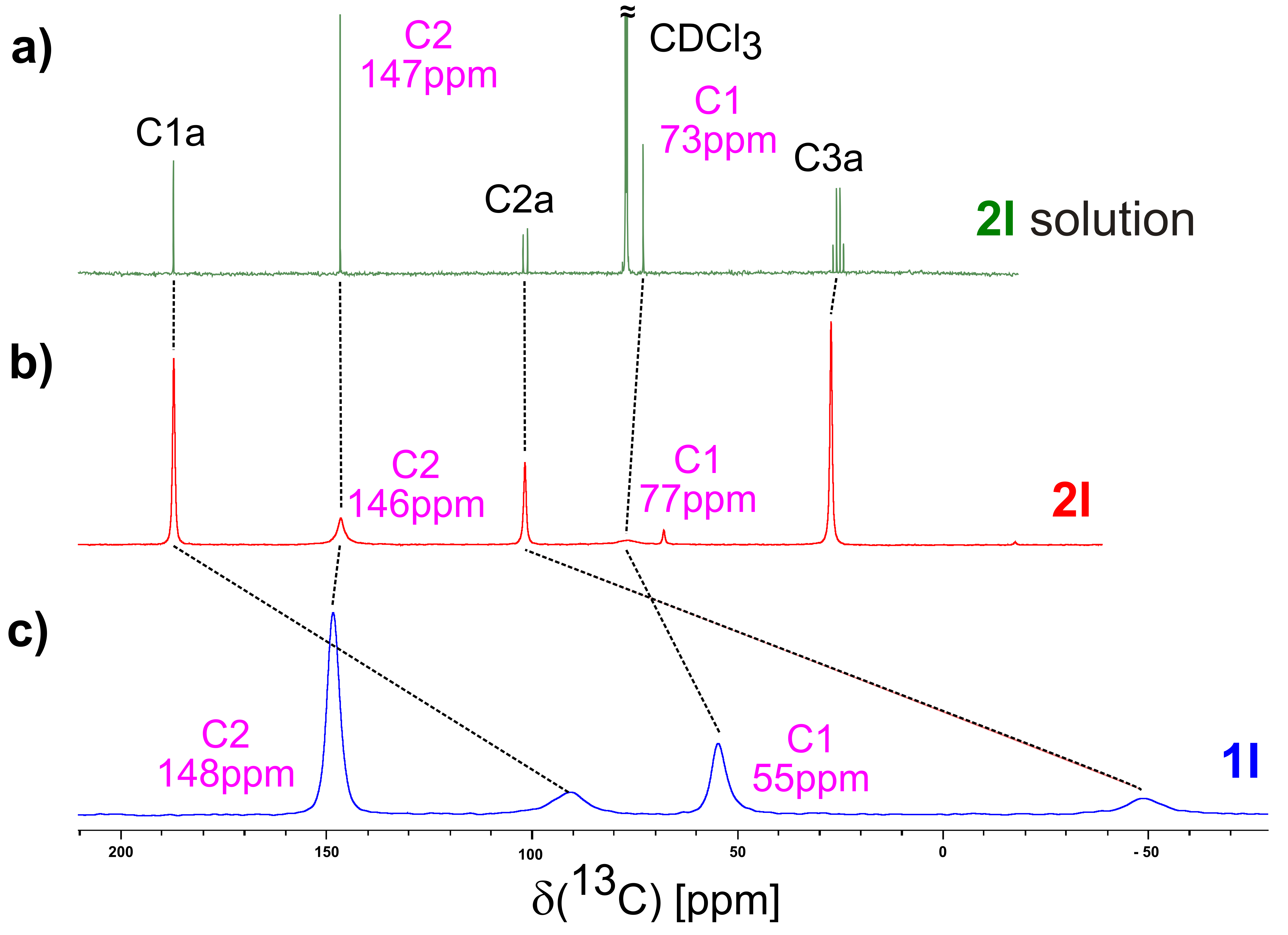

Supplement: SC-016-D5SC05769H-s001 [file SC-016-D5SC05769H-s001.zip › ESI Figures/FigureS7.png]

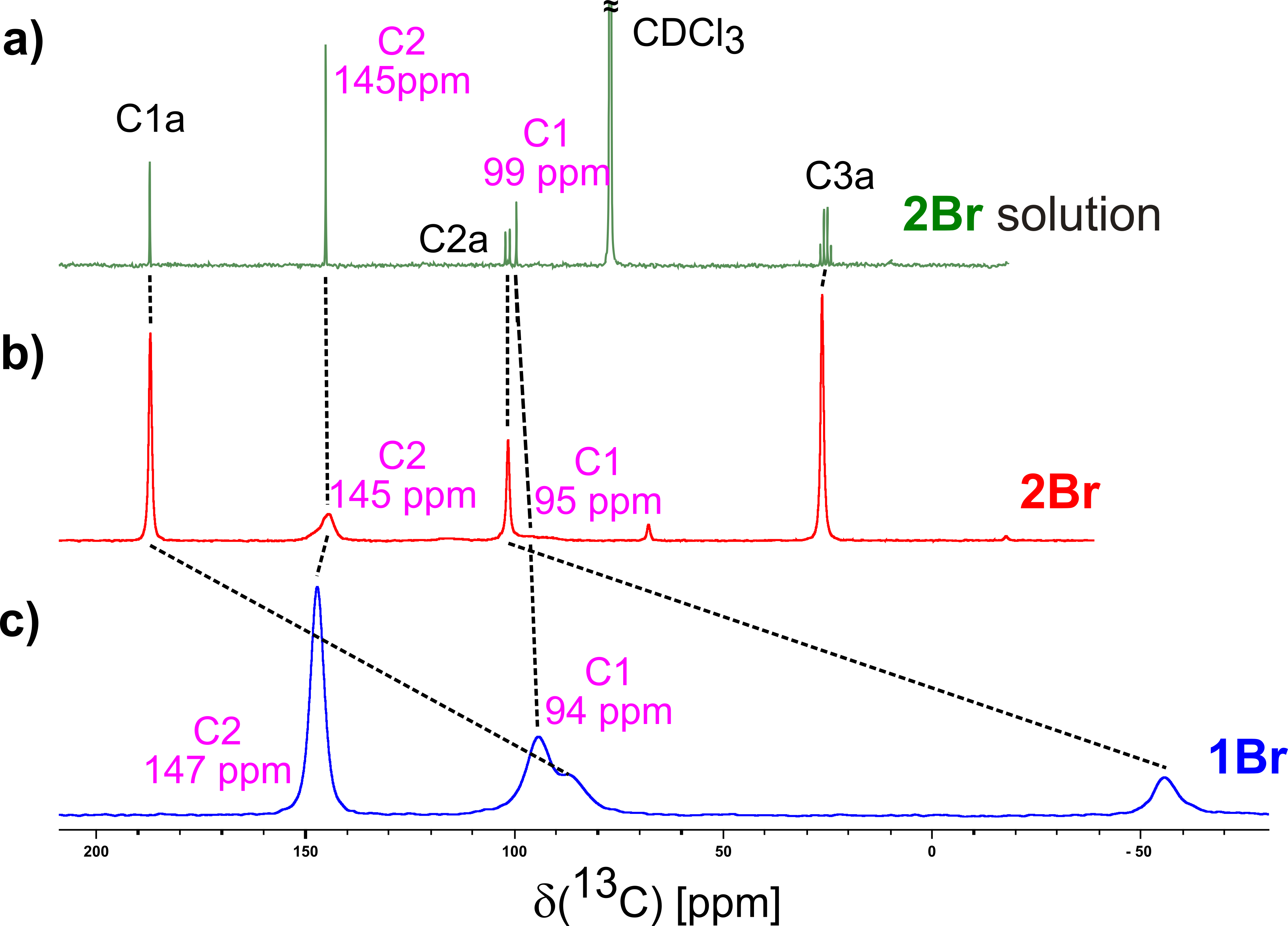

Supplement: SC-016-D5SC05769H-s001 [file SC-016-D5SC05769H-s001.zip › ESI Figures/FigureS8.png]

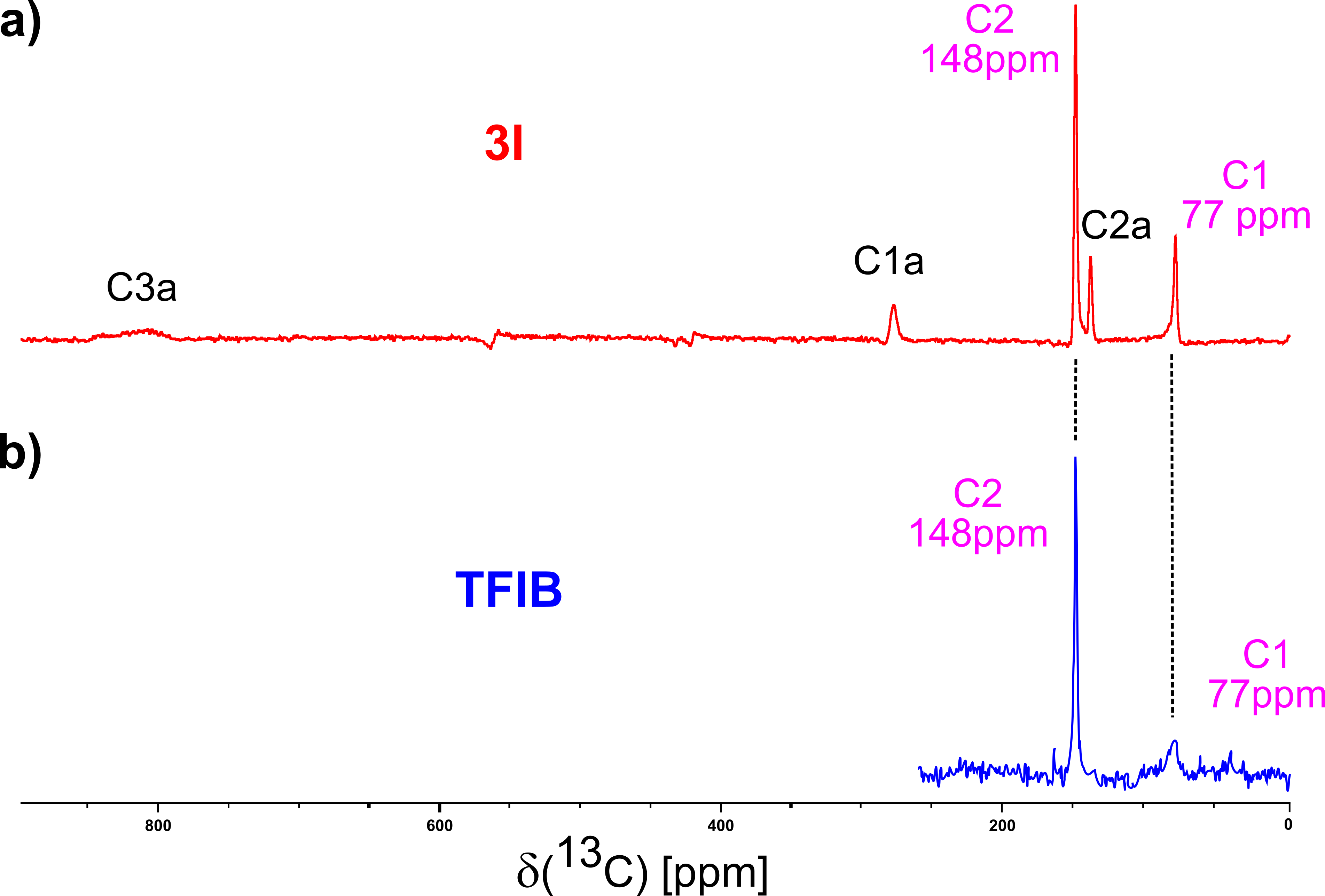

Supplement: SC-016-D5SC05769H-s001 [file SC-016-D5SC05769H-s001.zip › ESI Figures/FigureS9.png]
