# Supplementary material for: Supramolecular covalency of halogen bonds revealed by NMR contact shifts in paramagnetic cocrystals
Source: Chem Sci. 2025 Oct 2;16(43):20239–44. doi: 10.1039/d5sc05769h (PMC12516516; doi:10.1039/d5sc05769h)
Supplement: SC-016-D5SC05769H-s002 [file SC-016-D5SC05769H-s002.pdf]

Supplementary Information (SI) for Chemical Science.
This journal is © The Royal Society of Chemistry 2025

Supporting Information:

Supramolecular covalency of halogen bond revealed by NMR contact shift in paramagnetic cocrystals

Anagha Sasikumar,[†] Jan Novotný,[†,‡,¶] Jan Chyba,[†,‡] Libor Kobera,[§] and Radek Marek[*,†,‡,¶]

†*CEITEC - Central European Institute of Technology, Masaryk University, Kamenice 5, CZ-62500 Brno, Czechia*

‡*Department of Chemistry, Faculty of Science, Masaryk University, Kamenice 5, CZ-62500 Brno, Czechia*

¶*National Center for Biomolecular Research, Faculty of Science, Masaryk University, Kamenice 5, CZ-62500 Brno, Czechia*

§*Institute of Macromolecular Chemistry, Czech Academy of Sciences, Heyrovského nám. 2, CZ-16200 Prague, Czechia*

E-mail: radek.marek@ceitec.muni.cz

1 Experimental Details

1.1 Materials

Copper(II) acetylacetonate (97%), nickel(II) acetylacetonate (95%), and palladium(II) acetylacetonate (99%) were obtained from Merck. 1,4-Diiodotetrafluorobenzene (tfib) (97%) and 1,4-dibromotetrafluorobenzene (tfbb) (98%) were purchased from ABCR GmbH and BLD-pharm, respectively. All solvents, methanol, ethanol (both Lachner), chloroform (Penta), were of p.a. grade and were used as received. Deuterated chloroform (99,8% D) stabilized with silver foils was obtained from Eurisotop and used for the preparation of NMR samples in the form of solution.

The cocrystals were prepared according to known procedures by slow evaporation of the solution prepared by mixing the corresponding $[M^{II}(acac)_2]$ (M= Cu, Pd) complex with tfib (tfbb) and $[Ni^{II}(acac)_2(H_2O)_2]$ complex with tfib in a 1:1 molar ratio.[S1,S2]

1.2 Single-crystal X-ray Diffraction

The molecular structures of the grown monocrystals were verified by single-crystal X-ray diffraction. The diffraction data were collected at 120 K. The identity with known crystal structures CCDC no. 1830010 and 1830016 was determined for $[M^{II}(acac)_2]\cdot$tfib (M = Cu, Pd) system. XRD revealed the polymorphic form of $[Cu^{II}(acac)_2]\cdot$tfbb and $[Ni^{II}(acac)_2(H_2O)_2]$ $\cdot$tfib cocrystals to previously published X-ray structures CCDC no. 1858536 and 1858531. Selected crystallographic data and structural refinement parameters for **1Br** (CCDC no. 2472007, **Figure S1**), **2Br** (CCDC no. 2472009, **Figure S2**), and **3I** (CCDC no. 2472008, **Figure S3**) are listed in **Table S1**. The crystal packing of systems **1Br** (**Figure S4**), **2Br** (**Figure S5**), and **3I** (**Figure S6**) is also shown.

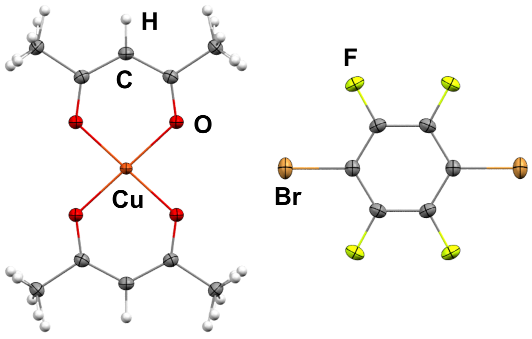

Figure S1: Supramolecular structure of 1Br determined by X-ray diffraction analysis

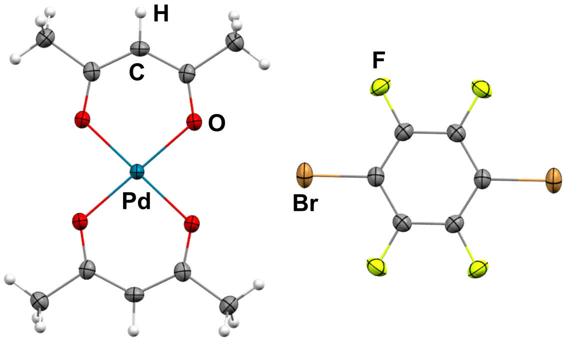

Figure S2: Supramolecular structure of 2Br determined by X-ray diffraction analysis

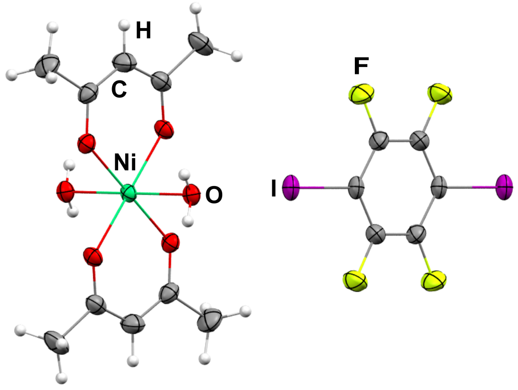

Figure S3: Supramolecular structure of 3I determined by X-ray diffraction analysis

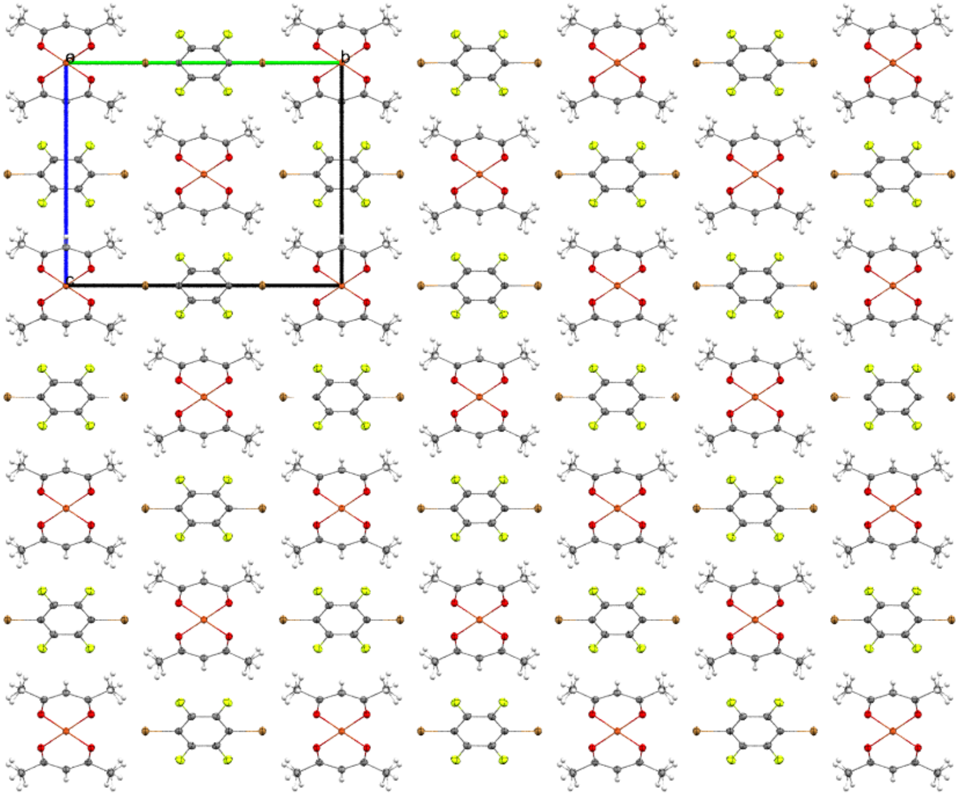

Figure S4: Crystal packing for system **1Br**.

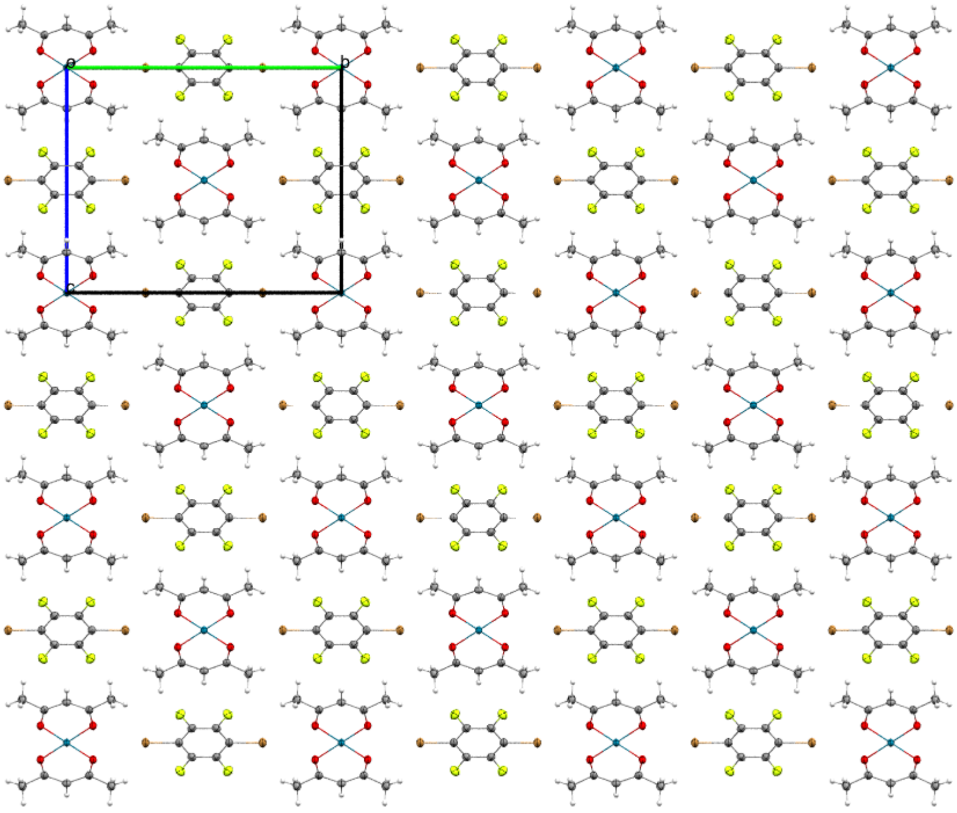

Figure S5: Crystal packing for system **2Br**.

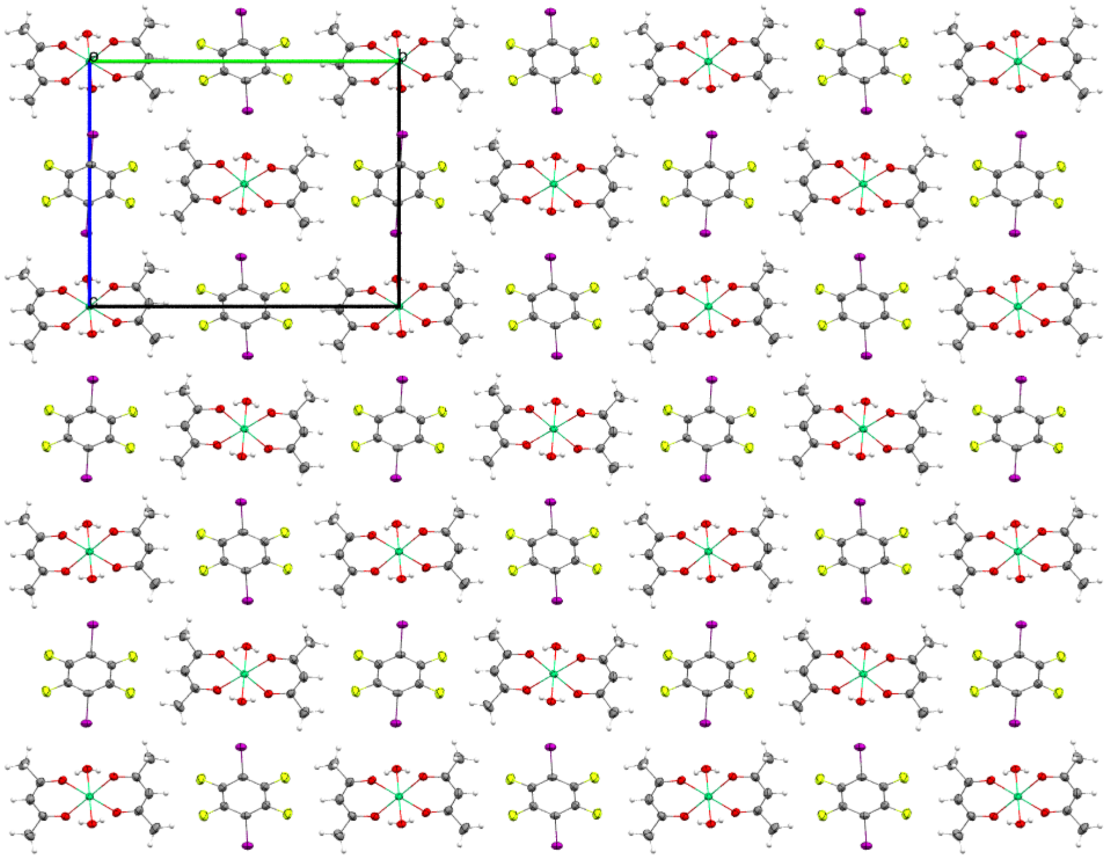

Figure S6: Crystal packing for system **3I**.

Table S1: Selected Crystallographic Data for Compounds **1Br**, **2Br**, and **3I**.

	**1Br**	**2Br**	**3I**
CCDC No	2472007	2472009	2472008
chemical formula	$C_{16}H_{14}Br_2CuF_4O_4$	$C_{16}H_{14}Br_2F_4O_4Pd$	$C_{18}H_{24}F_4I_2NiO_6$
formula weight	569.63	612.49	724.88
crystal system	Monoclinic	Monoclinic	Monoclinic
space group	*I 2/m*	*I 2/m*	*P 21/c*
a (Å)	4.6560(4)	4.73648(19)	4.67010(10)
b (Å)	15.4204(4)	15.3598(5)	17.3042(5)
c (Å)	12.4865(3)	12.6168(5)	13.7029(4)
α (deg)	90	90	90
β (deg)	93.929(4)	94.786(4)	90.653(2)
γ (deg)	90	90	90
V (Å^3)	894.39(8)	914.69(6)	1107.29(5)
Z	1	2	2
$D_{\text{calcd.}}$ (g cm^{-3})	1.058	2.224	2.174
μ (mm^{-1})	3.771	13.875	23.772
measured/unique reflections	4004/975	4130/979	13327/2325
data/parameters	975/86	979/67	2325/142
R_1/wR_2 [$I > 2\sigma(I)$]	0.0219/0.0639	0.0440/0.1310	0.0271/0.0757
R_1/wR_2 [all data]	0.0221/0.0641	0.0441/0.1311	0.0276/0.0761
GoF	0.829	1.150	1.087
$\Delta\rho_{\max}/\Delta\rho_{\min}$ (e Å^{-3})	0.38/-0.48	1.34/1.50	1.79/-0.74

1.3 NMR spectroscopy

Solid-state ^{13}C NMR spectra of **1I**, **1Br**, and **3I** were recorded at 16.4 T using a Bruker AVANCE Neo NMR spectrometer. The 1.3 mm magic-angle-spinning (MAS) probe was used for the experiment at a Larmor frequency of $\nu(^{13}\text{C}) = 176.110$ MHz. The paramagnetic samples require the use of the very fast magic-angle-spinning (VF/MAS) NMR approach.[S3] Therefore, the ^{13}C VF/MAS NMR experiments were conducted at a MAS rate of 50 kHz. The rotor synchronized spin-echo[S4] pulse sequence ($\pi/2$–t_1–π–aq) with one loop was used for all samples. The spectra were recorded using two short high-power adiabatic pulses (SHAPs)[S5] swept through 200 kHz and pulse duration of 30 μs, a recycle delay of 0.5 s, and 185k scans. The NMR experiments were performed at a temperature of 298 K and a temperature calibration was performed to compensate for the frictional heating of the samples.

The ^{13}C CP/MAS NMR spectra of **2I** and **2Br** were recorded on a Bruker 500 MHz Avance III HD spectrometer at Larmor frequency $\nu(^{13}\mathrm{C})$ = 125.784 MHz, using a 3.2-mm double resonance ^{1}H/^{19}F-X NMR probe with the diplexer filter to separate ^{1}H and ^{19}F NMR signals acquired on two receivers. The experiments were performed at a spinning frequency of 15 kHz and 8k scans. The sample was recorded with a 6 ms ^{1}H-^{13}C spin-lock and 6 s repetition delay. Simultaneous ^{1}H SPINAL64[S6] and ^{19}F Pi-Pulse[S7] decouplings, were used for the elimination of dipolar interactions during acquisition of ^{13}C CP/MAS NMR spectrum. The analyzed samples were placed into the ZrO_2 rotors and all NMR experiments were performed at 295 K.

The solid-state ^{13}C NMR shifts were calibrated using α-glycine as an external standard and referenced to 176.03 ppm (C=O). All spectra were processed using the TopSpin 3.2 pl5 software package.

The solution-state ^{13}C(^{19}F-decoupled) NMR spectra of **2Br** and **2I** were measured on a Bruker Avance Neo HD spectrometer at Larmor frequency $\nu(^{13}\mathrm{C})$ = 125 MHz at 298.2 K. NMR samples were prepared by dissolving 5-10 mg of cocrystals in 0.5 mL of $CDCl_3$. The NMR signal of the solvent $\delta(^{13}\mathrm{C})$ = 77.26 ppm was used as a secondary reference, and the NMR shifts are reported relative to TMS.

1.3.1 Experimental ^{13}C NMR spectra

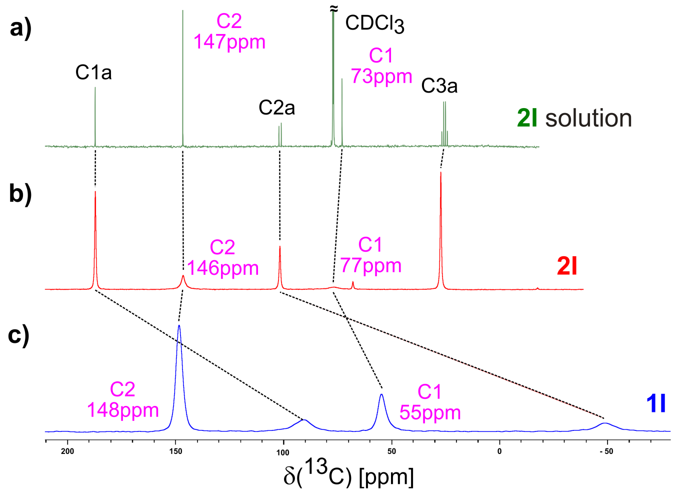

Figure S7: ^{13}C NMR spectra obtained for systems (c) **1I** and (b) **2I** in solid state, and (a) **2I** in $CDCl_3$ solution at 298.2 K.

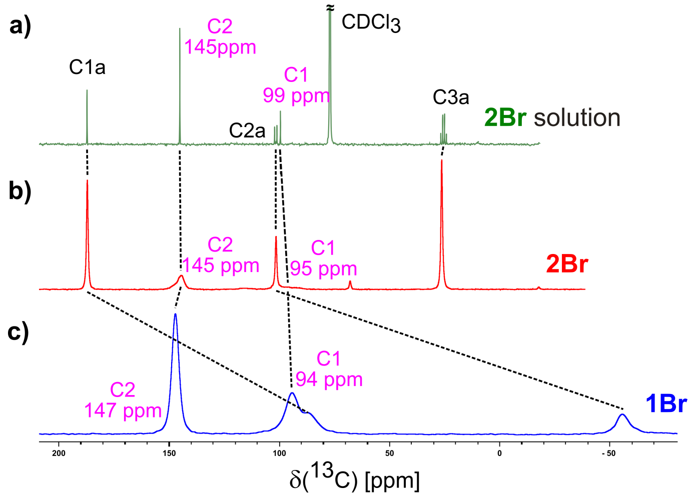

Figure S8: ^{13}C NMR spectra obtained for systems (c) **1Br** and (b) **2Br** in solid state, and (a) **2Br** in $CDCl_3$ solution at 298.2 K.

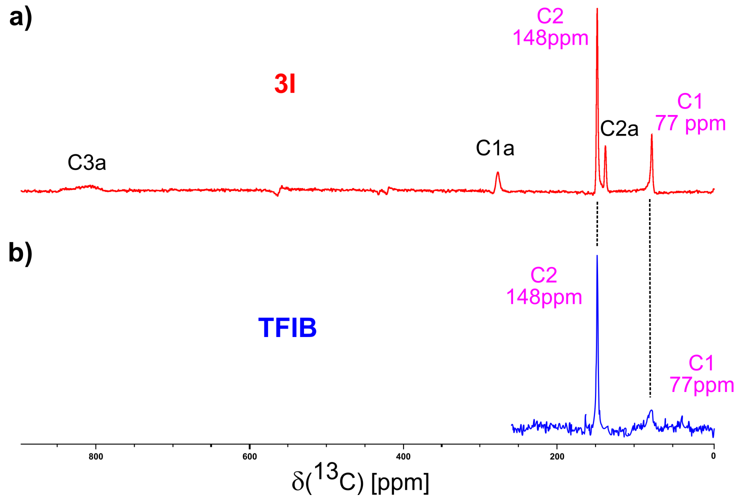

Figure S9: ^{13}C NMR spectra obtained for (a) system **3I** in solid state and (b) TFIB in $CDCl_3$ solution at 298.2 K.

2 Computational Details

The structures of the systems described here are taken from previous XRD studies with CCDC nos. 1858530 for **1I**,[S1] 1830016 for **2I**,[S2] 1858536 for **1Br**,[S1] and 1858531 for **3I**.[S1] The structure of **2Br** (CCDC no. 2472009) was determined in this work; see Section 1.2. The positions of the hydrogens in the supramolecular systems were optimized using density functional theory, while the heavier elements were fixed at their respective crystallographic positions. The ↑···↑ spin state was considered for system **1**. Optimization was performed using a hybrid GGA functional PBE0 and a triple zeta quality basis set (TZ2P) as implemented in the ADF 2024 program.[S8] Relativistic corrections were taken into account using the 1c or 2c ZORA (Zero-Order Regular Approximation) Hamiltonian.

2.1 Optimization of system size and computational method

The model size that best represents the cocrystal was selected by evaluating the hyperfine shifts for three systems: $\mathbf{1I}_{small}$, **1I**, and $\mathbf{1I}_{large}$, as shown in **Figure S10**. The hyperfine shifts converge for system sizes beyond **1I**, indicating that **1I** provides a sufficiently accurate representation as indicated in **Figure S11**. Therefore, system **1I** was chosen as the representative model size for all production NMR shift calculations.

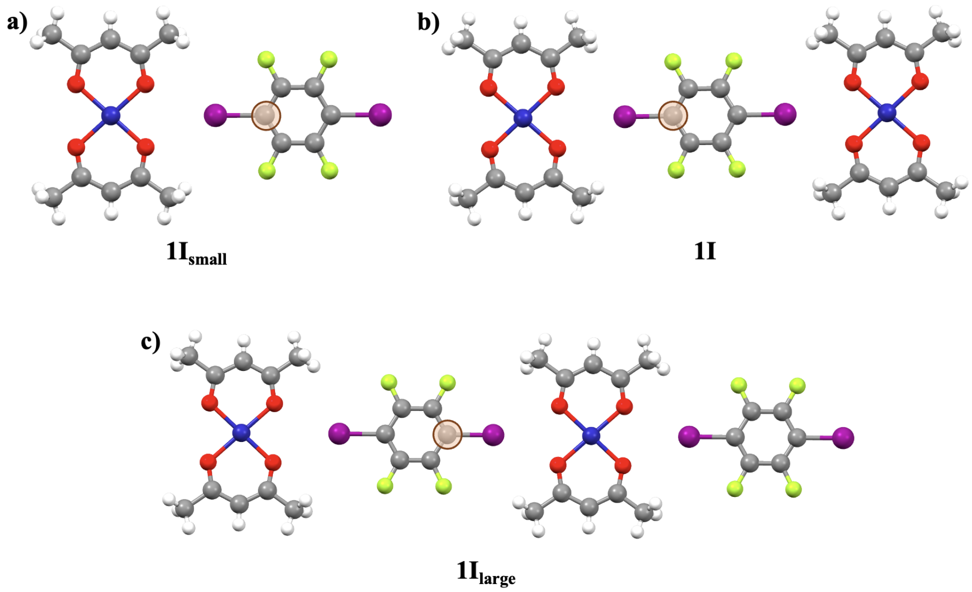

Figure S10: Model systems of various sizes: (a) **1I**$_{small}$ with one pair of $Cu(acac)_2$ and $C_6F_4X_2$, (b) **1I** with one $C_6F_4X_2$ between two $Cu(acac)_2$ molecules and (c) **1I**$_{large}$ with two pairs of $Cu(acac)_2$ and $C_6F_4X_2$. Hyperfine shifts of circled C1 atoms in each system are considered for evaluation.

Hyperfine shifts were further evaluated using the hybrid PBE0 functional with a varying degree of exact-exchange (Hatree-Fock) admixture as shown in **Figure S12**. Although the best agreement with the experiment would be achieved for the most indicative NMR shift of C1 at 40% of the ExEx admixture, the PBE50 approach with 50% ExEx admixture was chosen. This choice further reduces the delocalization error in the PBE0 approach while providing decent accuracy. Note that here the spin contamination is negligible in all cases (25-80% ExEx admixture).

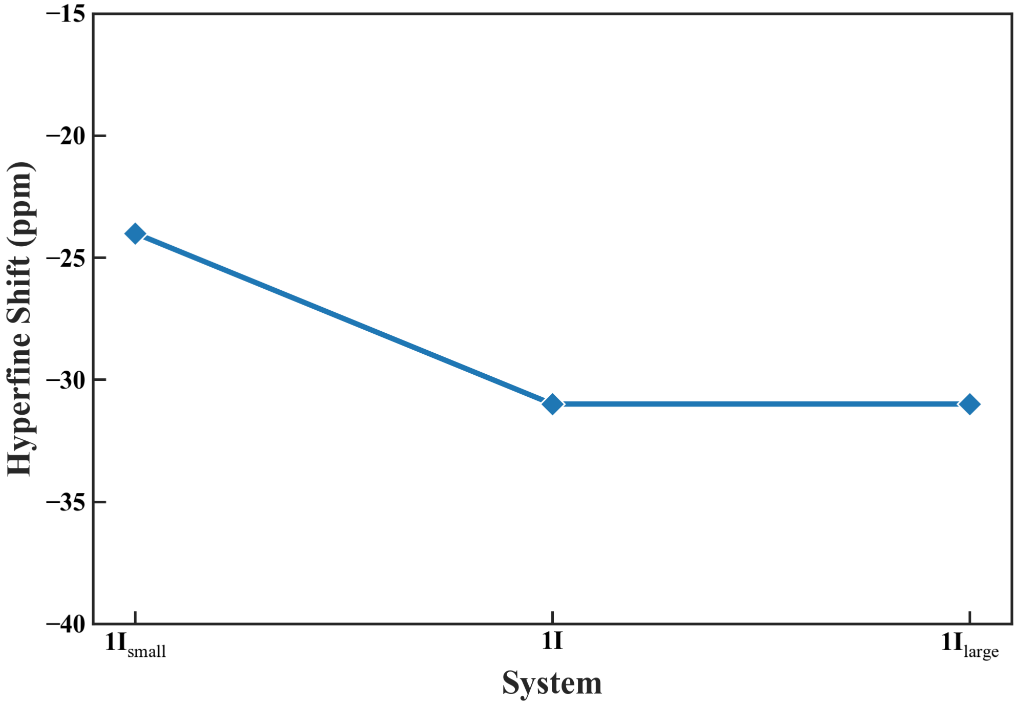

Figure S11: Hyperfine ^{13}C NMR shift of atom C1 (indicated in **Figure S10**) calculated for different model sizes (PBE50, TZ2P, 298 K). The NMR shift is shown for the parallel orientation of spins ($\uparrow \cdots \uparrow$) in System **1I** and $\mathbf{1I}_{large}$.

2.2 DFT calculation of NMR shifts

For the open-shell systems **1I**, **1Br**, and **3I**, following the geometry optimization, computations of the orbital and hyperfine shifts were performed. For hyperfine shifts, the EPR parameters, **A**-tensor at the 1-component (1c) scalar-relativistic ZORA and the 2-component (2c) spin-orbit (SO) ZORA level, and **g**-tensor at the 2-component SO-ZORA level were calculated. The NMR shifts (δ) corresponding to the orbital contributions to the NMR shifts (δ^{orb}) were calculated for the diamagnetic analogs of systems **1I**, **1Br**, and **3I** obtained by replacing $Cu^{2+}(\uparrow)$ with $Ni^{2+}(\uparrow\downarrow)$ in **1I** and **1Br**, and $Ni^{2+}(\uparrow\uparrow)$ with $Zn^{2+}(\uparrow\downarrow)$ in **3I**, keeping the geometry of the system intact. The calculations were performed using a PBE50 (PBE0 functional with 50% of the exact-exchange admixture) and the TZ2P set of bases. The expectation value for the spin are checked to be within the error limit to avoid possible spin contamination.

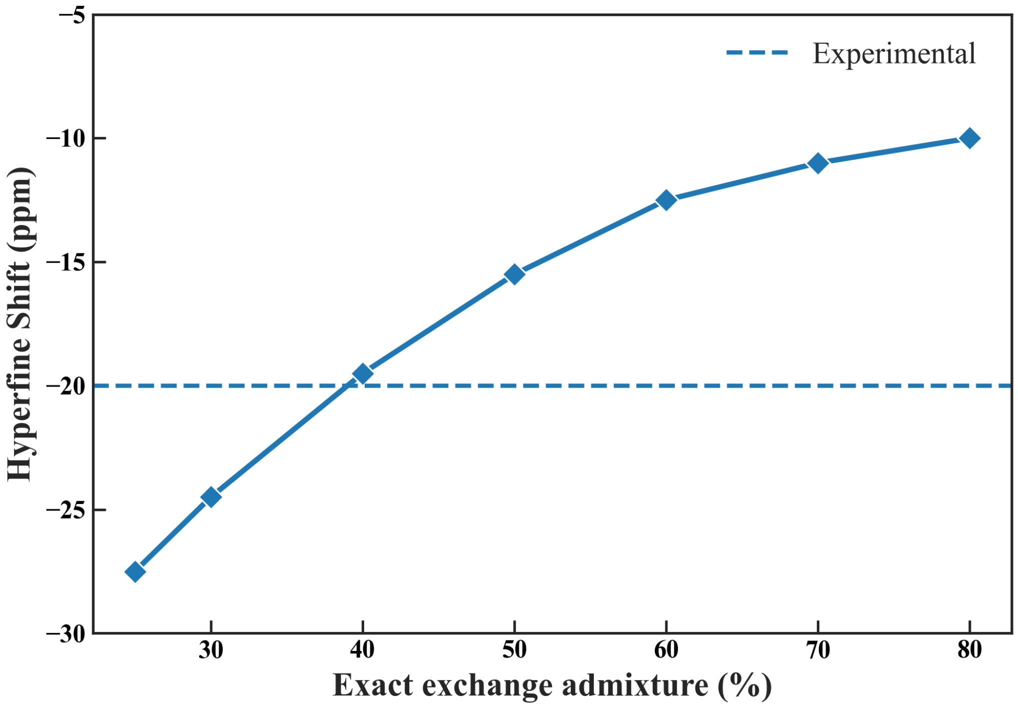

Figure S12: Hyperfine ^{13}C NMR shift of C1 in system **1I** calculated for varying degree of exact-exchange (Hartree-Fock) admixture. The dashed line represents the experimental value.

The pNMR chemical shifts and the orbital, Fermi contact, and pseudo-contact contributions to it were evaluated using the *PNMRShift* program at 298 K.[S9,S10] Note that here the σ tensor and the **A**-tensor are evaluated at the corresponding level (1c ZORA or 2c SO-ZORA), whereas the **g**-tensor is always calculated at the 2c SO-ZORA level. The NMR shifts of the equivalent carbons are averaged.

2.3 Effect of different spin combinations

For system **1I**, parallel spin state ($\uparrow \cdots \uparrow$) and antiparallel spin state ($\uparrow \cdots \downarrow$) are possible for the two Cu($\uparrow$) centers. The exchange coupling (J) is negligible in this case,[S11] causing the two spin states to be of 50% population each. For the parallel state ($\uparrow\uparrow$), the hyperfine shifts of the two equivalent carbon atoms C1 are observed to be the same. For the antiparallel state ($\uparrow\downarrow$), the hyperfine shifts are equal in magnitude for the two atoms C1, but opposite

in sign. For the crystal, averaging over the two equally populated spin states, the hyperfine NMR shift is divided by 2 (see Tables S2 and S3).

2.4 Analyses of halogen bond

At first, the bonding in system **1I** and **1Br** was analysed using energy decomposition analysis (EDA) implemented in ADF package.[S12] The calculation was performed at the scalar-relativistic ZORA level using PBE50/TZ2P. Dispersion correction was applied using a Grimme D3 (BJ) method.[S13] The total interaction energy was decomposed according to the following equation:

$$\Delta E_{\text{int}} = \Delta E_{\text{Es}} + \Delta E_{\text{Pauli}} + \Delta E_{\text{Orbital}} + \Delta E_{\text{Disp}} \quad (1)$$

NOCV analysis was performed and the important channels were identified. The deformation densities were mapped for the four NOCV channels involving charge transfer from lone pairs of the four oxygen atoms to the two vacant antibonding C-I orbitals. The electron densities were added to obtain the total EDD corresponding to the two bifurcated halogen bonds. The stabilization energy contribution of these channels are evaluated.

To further analyze the halogen bond, a smaller model system consisting of one $Cu(acac)_2$ and one organic moeity $C_6F_4X_2$ was considered. The same level of theory as described above was used. The analysis of the delocalization indices (DI) using quantum theory of atoms in molecules (QTAIM) was performed as implemented in the ADF 2024 package.

3 Calculated NMR Shifts

Table S2: ^{13}C NMR shifts (in ppm) for systems **1I-3I** and **1Br** calculated at the scalar-relativistic (1c ZORA) level using the PBE50/TZ2P approach.

System	Atom	$\delta^{orb\,a}$	$\delta^{HF\,b}$	Total
**1I**	C1	109	−16	93
	C2	142	+1	143
	C1a	213	+3	216
	C2a	112	+113	225
	C3a	24	+833	857
**1Br**	C1	110	−9	101
	C2	139	+1	140
	C1a	208	-23	185
	C2a	110	+116	226
	C3a	23	+897	920
**2I**	C1	105	–	105
	C2	143	–	143
	C1a	196	–	196
	C2a	99	–	99
	C3a	19	–	19
**2I**	C1	118	–	118
	C2	146	–	146
	C1a	196	–	196
	C2a	97	–	97
	C3a	19	–	19
**3I**	C1	103	−2	101
	C2	140	0	140
	C1a	195	+116	311
	C2a	90	+40	130
	C3a	20	+966	986

[a] To calculate δ^{orb} at the ZORA level, $Cu^{2+}(\uparrow)$ was replaced by $Ni^{2+}(\uparrow\downarrow)$ in **1I** and **1Br**, and $Ni^{2+}(\uparrow\uparrow)$ was replaced by $Zn^{2+}(\uparrow\downarrow)$ in **3I**.

[b] The FC values for C1 and C2 in **1I**, **1Br**, and **3I** were obtained by averaging δ^{HF} for two spin states, parallel and antiparallel. Note that the hyperfine shifts for C1a, C2a, and C3a are not averaged as they are all affected by *ipso* $Cu^{2+}(\uparrow)$ center.

Table S3: ^{13}C NMR shifts (ppm) for systems **1I-3I** and **1Br** calculated at the spin-orbit (2c SO-ZORA) level using the PBE50/TZ2P and obtained experimentally.

System	Atom	$\delta^{orb\,a}$	$\delta^{HF\,b}$	Total	Experimental
**1I**	C1	78	−16	62	55
	C2	147	+1	148	146
	C1a	208	−3	205	91
	C2a	112	−114	−2	−49
	C3a	10	+831	841	–
**1Br**	C1	102	−9	93	94
	C2	143	0	143	147
	C1a	205	−23	182	87
	C2a	110	−116	−6	−56
	C3a	10	+894	904	–
**2I**	C1	76	–	76	76
	C2	147	–	147	146
	C1a	195	–	195	187
	C2a	100	–	100	102
	C3a	17	–	17	26
**2Br**	C1	104	–	104	95
	C2	145	–	145	145
	C1a	194	–	194	187
	C2a	98	–	98	102
	C3a	17	–	17	26
**3I**	C1	73	−2	71	77
	C2	145	0	145	148
	C1a	194	+122	316	277
	C2a	91	+41	132	136
	C3a	21	+970	991	805

[a] To calculate δ^{orb} at the SO-ZORA level, $Cu^{2+}(\uparrow)$ was replaced by $Ni^{2+}(\uparrow\downarrow)$ in **1I** and **1Br**, and $Ni^{2+}(\uparrow\uparrow)$ was replaced by $Zn^{2+}(\uparrow\downarrow)$ in **3I**.

[b] The FC values for C1 and C2 in **1I**, **1Br**, and **3I** were obtained by averaging δ^{HF} for two spin states, parallel and antiparallel. Note that the hyperfine shifts for C1a, C2a, and C3a are not averaged as they are all affected by *ipso* $Cu^{2+}(\uparrow)$ center.

Table S4: Comparison of ^{13}C NMR shifts (ppm) for systems **1I-3I** and **1Br** calculated at the spin-orbit (2c SO-ZORA) level using the PBE50/TZ2P and PBE0/TZ2P.

System	Atom	δ^{orb}_{PBE50}	δ^{HF}_{PBE50}	δ^{tot}_{PBE50}	δ^{orb}_{PBE0}	δ^{HF}_{PBE0}	δ^{tot}_{PBE0}	δ^{exp}
**1I**	C1	78	−16	**62**	82	−28	**54**	**55**
	C2	147	+1	**148**	148	+1	**149**	**146**
	C1a	208	−3	**205**	196	−87	**109**	**91**
	C2a	112	−114	**−2**	108	−165	**−57**	**−49**
	C3a	10	+831	**841**	19	+1340	**1359**	−
**1Br**	C1	102	−9	**93**	104	−16	**88**	**94**
	C2	143	0	**143**	145	+1	**146**	**147**
	C1a	205	−23	**182**	192	−97	**95**	**87**
	C2a	110	−116	**−6**	107	−173	**−66**	**−56**
	C3a	10	+894	**904**	17	+1448	**1465**	−
**2I**	C1	76	−	**76**	80	−	**80**	**76**
	C2	147	−	**147**	149	−	**149**	**146**
	C1a	195	−	**195**	189	−	**189**	**187**
	C2a	100	−	**100**	103	−	**103**	**102**
	C3a	17	−	**17**	19	−	**19**	**26**
**3I**	C1	73	−2	**71**	77	+1	**78**	**77**
	C2	145	0	**145**	146	0	**146**	**148**
	C1a	194	+122	**316**	192	+158	**350**	**277**
	C2a	91	+41	**132**	94	+136	**230**	**136**
	C3a	21	+970	**991**	22	+1472	**1494**	**805**

4 Models of Halogen Bonded Systems

To comment on the nature of the halogen bond (XB), we additionally considered three models of varying XB strength, namely, (i) *Model 1*: complex of ammonia and iodofluride, (ii) *Model 2*: complex of $Cu(acac)_2$ with iodofluride, and (iii) *Model 3*: complex of $Cu(acac)_2$ with trifluoroiodomethane as shown in **Figure S13**. The degree of covalency of the model complexes, as indicated by their delocalization indices, is compared to system **1I**. The DIs for *Model 1*, *Model 2*, and *Model 3* are evaluated to be 0.44, 0.22, and 0.14, respectively, compared to 0.12 of system **1I**. This is in agreement with the reduced coupling constants K_{N-I} 1059.4 10^{19} kg m^{-2} s^{-2} A^{-2} for *Model1*, K_{O-I} 200.1 10^{19} kg m^{-2} s^{-2} A^{-2} for *Model2* and K_{O-I} 105.6 10^{19} kg m^{-2} s^{-2} A^{-2} for *Model3* compared to K_{O-I} 154.7 10^{19} kg m^{-2} s^{-2} A^{-2} for system **1I**. The hyperfine ^{13}C NMR shift calculated at 2c DFT level for CF_3I molecule in *Model 3* is −45 ppm compared to −31 ppm for the closest C1 in the system **1I**.

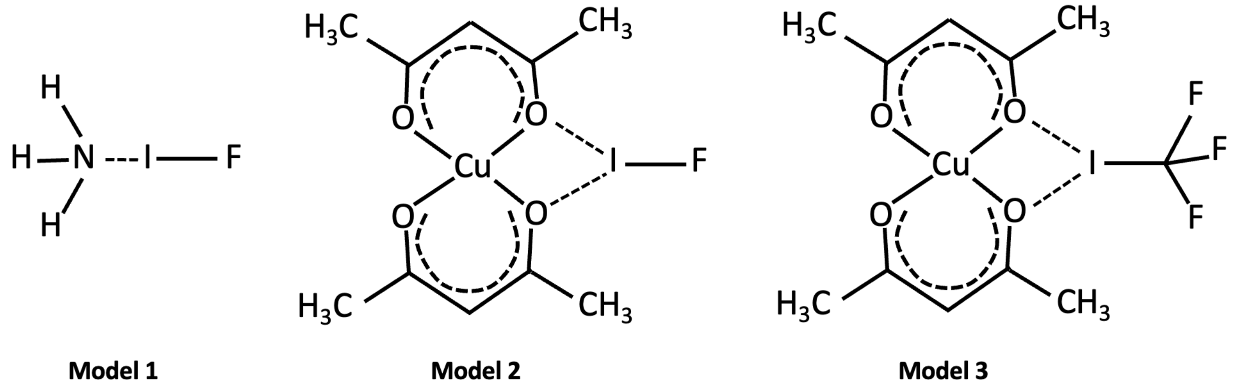

Figure S13: Halogen bonded models used to compare the DIs and strength of the halogen bond with system **1I**. *Model 1*: complex of ammonia with iodofluride, *Model 2*: complex of $Cu(acac)_2$ with iodofluride, and *Model 3*: complex of $Cu(acac)_2$ with trifluoroiodomethane.

5 Spin Density for System 3I

To understand the spin transmission mechanism in detail and analyse the role of the delocalisation pathway through H_2O (O_{ax}) and polarisation pathway through *acac*s (O_{eq}) we considered three configurations: (a) system **3I**, (b) **3I** with tfib tilted such that the angle between I, O_{ax} and Cu is 120°, (c) 3I with tfib rotated by 120° around an axis passing through O_{ax} and Cu such that tfib is between the Os of an acac ligand. The interaction of C1 with the *acac* ligands is minimized in (b) and H_2O is minimized in (c). The spin density distribution (**Figure S14**) indicates an increased α-spin density at C1 in case of (b) and a β-spin density in the case of (c). This is refelcted by the hyperfine shifts +12 and −9 ppm for (b) and (c) compared to −3 ppm for system 3I (a).

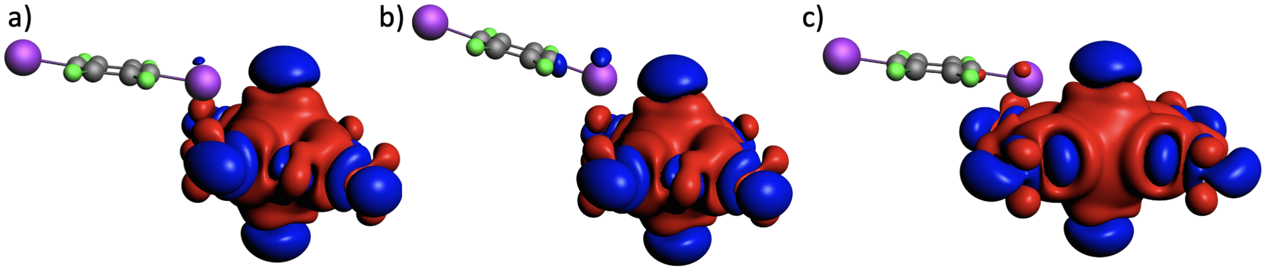

Figure S14: Visualization of spin density for system (a) **3I**, (b) **3I** with tfib tilted, and (c) 3I with tfib rotated at an isovalue of 0.00001 a.u.

6 Summary of NMR Shift Calculations and Halogen Bond Analyses

Table S5 summarizes the hyperfine NMR shift calculations and various analyses of halogen bonds in systems **1-3I**, **1-2Br**, and the diamagnetic analog **3I-Zn**, obtained by replacing Ni(II) with Zn(II) in **3I**. The NMR shift values are reported in ppm, the supramolecular energy terms (ΔE_{orb}, ΔE_{int}) in kcal mol^{-1}, and the reduced coupling constants (K) in 10^{19} kg m^{-2} s^{-2} A^{-2}. O_{eq} an O_{ax} in system **3I** and **3I-Zn** represents the O atoms of acac and H_2O, respectively.

The higher values of DI, ΔE_{int} and reduced coupling constant K indicates a stronger halogen bond in case of **1I** and **2I** compared to **1Br** and **2Br** in agreement with the higher hyperfine shifts for the former. In the case of **3I**, despite having comparable interaction energies to system **1I** and **1Br**, the lower hyperfine shift can be attributed to multiple partly compensating spin transmission pathways.

Table S5: Hyperfine ^{13}C NMR shifts, delocalization indices, interaction energies, and reduced coupling constant for systems **1-3**.

System	$\delta^{HF\,a}$	DI	ΔE_{orb}	ΔE_{int}	K	Spin Population at O
**1I**	-16	0.12	-1.9	-7.9	–	0.05
**1Br**	-9	0.10	-1.8	-6.5	–	0.05
**2I**	–	0.16	-2.5	-7.9	O-C1: 5.1<br>I-C1: -455.5<br>O-I: 154.7	–
**2Br**	–	0.12	-2.1	-6.7	O-C1: 2.8<br>Br-C1: -262.8<br>O-Br: 56.8	–
**3I**	-2	O_{eq}:0.10<br>O_{ax}:0.01	-3.2	-7.0	–	O_{eq}: 0.03<br>O_{ax}: 0.02
**3I-Zn**	–	–	–	–	O_{ax}-C1:1 0.9<br>O_{ax}-I: 306.9<br>O_{eq}-C1: 0.4<br>O_{eq}-I: 45.6<br>I-C: -475.4	–

[a] The hyperfine shifts for C1 and C2 in **1I**, **1Br**, and **3I** were obtained by averaging δ^{HF} for two spin states, parallel and antiparallel.

6.1 Electron deformation density for system $\mathbf{1I}_{small}$

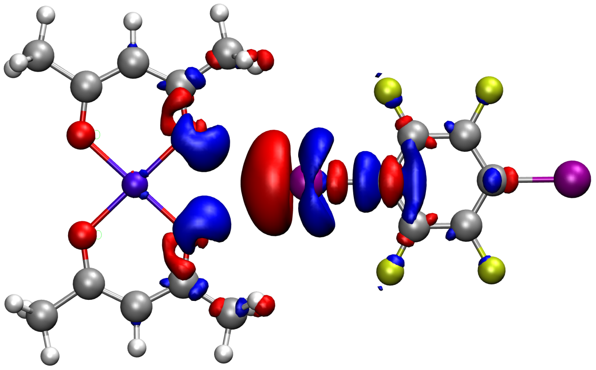

Figure S15: Electron deformation density (EDD) calculated for system $\mathbf{1I}_{small}$ (isovalue 0.0005 au). Concentration and depletion of electron density is shown in blue and red, respectively.

6.2 Comparison of interaction energies (ΔE_{int}) of the halogen bonds in 1I and 1Br with previously calculated values

Table S6: Energies (kJ mol^{-1}) of the halogen bonds for systems **1I** and **1Br**: A) Interaction energy ΔE_{int}^{A} calculated at the PBE50/TZ2P level in this work on the solid-state structures, compared to B) Bond energy ΔE_{bond}^{B} previously calculated at the M06/def2-TZVP level using the optimized structures.[S1] Note here that the bond energy in the previous work is expressed as ΔE_{bond} = -ΔE_{int} + ΔE_{def}, where ΔE_{def} is the monomer deformation (relaxation) energy.

System	ΔE_{int}^{A}	ΔE_{bond}^{B}
**1I**	-33.42	30.67
**1Br**	-27.29	21.85

6.3 Natural bond orbital analysis

The second order stabilization energy ($\Delta E^{(2)}$) resulting from the charge transfer from the lone pair of oxygen in the $Cu(acac)_2$ to the σ^*_{C-I} orbital, orbital energy $E_{(j)} - E_{(i)}$, and their orbital overlap indicated by the non-diagonal matrix element ($F_{LP\rightarrow\sigma^*}$) were evaluated Here, only a pair of $Cu(acac)_2$ and $C_6F_4X_2$ was used for the calculation taking advantage of the symmetry of the system. A lower second order stabilization energy and orbital overlap

for **1Br** reassert the weaker halogen bonding for **1Br** compared to **1I**, suggesting weaker electron sharing in the former case.

Table S7: Second order stabilization energy $\Delta E^{(2)}$ (kcal mol^{-1}), orbital energies $E_{(j)} - E_{(i)}$ (a.u.), and orbital overlap $F_{(i,j)}$ (a.u.) evaluated for systems **1I** and **1Br** evaluated at PBE0 level.

System	Donor NBO	Acceptor NBO	$\Delta E^{(2)}$	$E_{(j)} - E_{(i)}$	$F_{(i,j)}$
**1I**	LP$_O$	σ^*_{I-C}	0.44	0.72	0.023
**1Br**	LP$_O$	σ^*_{Br-C}	0.25	0.76	0.018

References

(S1) Stilinović, V.; Grgurić, T.; Piteša, T.; Nemec, V.; Cinčić, D. Bifurcated and Monocentric Halogen Bonds in Cocrystals of Metal(II) Acetylacetonates with p-Dihalotetrafluorobenzenes. *Cryst. Growth Des.* **2019**, *19*, 1245–1256.

(S2) Rozhkov, A. V.; Novikov, A. S.; Ivanov, D. M.; Bolotin, D. S.; Bokach, N. A.; Kukushkin, V. Y. Structure-Directing Weak Interactions with 1,4-Diiodotetrafluorobenzene Convert One-Dimensional Arrays of [MII(acac)2] Species into Three-Dimensional Networks. *Cryst. Growth Des.* **2018**, *18*, 3626–3636.

(S3) Ishii, Y.; Wickramasinghe, N. P.; Chimon, S. A New Approach in 1D and 2D 13C High-Resolution Solid-State NMR Spectroscopy of Paramagnetic Organometallic Complexes by Very Fast Magic-Angle Spinning. *J. Am. Chem. Soc.* **2003**, *125*, 3438–3439.

(S4) Hahn, E. L. Spin Echoes. *Phys. Rev.* **1950**, *80*, 580–594.

(S5) Fast adiabatic pulses for solid-state NMR of paramagnetic systems. *Chem. Phys. Lett.* **2007**, *435*, 157–162.

(S6) An Improved Broadband Decoupling Sequence for Liquid Crystals and Solids. *J. Magn. Reson.* **2000**, *142*, 97–101.

(S7) Liu, S.-F.; Schmidt-Rohr, K. High-Resolution Solid-State 13C NMR of Fluoropolymers. *Macromolecules* **2001**, *34*, 8416–8418.

(S8) Baerends, E. J.; Aguirre, N. F.; Austin, N. D.; Autschbach, J.; Bickelhaupt, F. M.; Bulo, R.; Cappelli, C.; van Duin, A. C. T.; Egidi, F.; Fonseca Guerra, C.; Förster, A.; Franchini, M.; Goumans, T. P. M.; Heine, T.; Hellström, M.; Jacob, C. R.; Jensen, L.; Krykunov, M.; van Lenthe, E.; Michalak, A.; Mitoraj, M. M.; Neugebauer, J.; Nicu, V. P.; Philipsen, P.; Ramanantoanina, H.; Rüger, R.; Schreckenbach, G.;

Stener, M.; Swart, M.; Thijssen, J. M.; Trnka, T.; Visscher, L.; Yakovlev, A.; van Gisbergen, S. The Amsterdam Modeling Suite. *J. Chem. Phys.* **2025**, *162*, 162501.

(S9) Autschbach, J.; Srebro, M. Delocalization Error and "Functional Tuning" in Kohn–Sham Calculations of Molecular Properties. *Acc. Chem. Res.* **2014**, *47*, 2592–2602.

(S10) Autschbach, J.; Patchkovskii, S.; Pritchard, B. Calculation of Hyperfine Tensors and Paramagnetic NMR Shifts Using the Relativistic Zeroth-Order Regular Approximation and Density Functional Theory. *J. Chem. Theory Comput.* **2011**, *7*, 2175–2188.

(S11) Novotný, J.; Jeremias, L.; Nimax, P.; Komorovsky, S.; Heinmaa, I.; Marek, R. Crystal and Substituent Effects on Paramagnetic NMR Shifts in Transition-Metal Complexes. *Inorg. Chem.* **2021**, *60*, 9368–9377.

(S12) Baerends, E.; Ziegler, T.; Atkins, A.; Autschbach, J.; Bashford, D.; Baseggio, O.; Bérces, A.; Bickelhaupt, F.; Bo, C.; Boerritger, P.; others ADF2019, SCM, theoretical chemistry. *Vrije Universiteit:Amsterdam: The Netherlands* **2019**,

(S13) Grimme, S.; Ehrlich, S.; Goerigk, L. Effect of the damping function in dispersion corrected density functional theory. *J. Comput. Chem.* **2011**, *32*, 1456–1465.
